# Iconicity as Multimodal, Polysemiotic, and Plurifunctional

**DOI:** 10.3389/fpsyg.2022.808896

**Published:** 2022-06-13

**Authors:** Gabrielle Hodge, Lindsay Ferrara

**Affiliations:** ^1^Deafness Cognition and Language Research Centre, University College London, London, United Kingdom; ^2^College of Asia and the Pacific, Australian National University, Canberra, ACT, Australia; ^3^Department of Language and Literature, Norwegian University of Science and Technology, Trondheim, Norway

**Keywords:** iconicity, indexicality, gesture, semiotics, sign language, typology

## Abstract

Investigations of iconicity in language, whereby interactants coordinate meaningful bodily actions to create resemblances, are prevalent across the human communication sciences. However, when it comes to analysing and comparing iconicity across different interactions (e.g., deaf, deafblind, hearing) and modes of communication (e.g., manual signs, speech, writing), it is not always clear we are looking at the same thing. For example, tokens of spoken ideophones and manual depicting actions may both be analysed as iconic forms. Yet spoken ideophones may signal depictive and descriptive qualities via speech, while manual actions may signal depictive, descriptive, and indexical qualities via the shape, movement, and placement of the hands in space. Furthermore, each may co-occur with other semiotics articulated with the face, hands, and body within composite utterances. The paradigm of iconicity as a single property is too broad and coarse for comparative semiotics, as important details necessary for understanding the range of human communicative potentialities may be masked. Here, we draw on semiotic approaches to language and communication, including the model of language as signalled via describing, indicating and/or depicting and the notion of non-referential indexicality, to illustrate the multidimensionality of iconicity in co-present interactions. This builds on our earlier proposal for analysing how different methods of semiotic signalling are combined in multimodal language use. We discuss some implications for the language and communication sciences and explain how this approach may inform a theory of biosemiotics.

## Introduction

Iconicity is generally defined as ‘fundamentally about resemblance’, whereby ‘just like paintings can resemble what they depict, so linguistic signs can look and sound like what they mean in various ways and to varying degrees’ (Dingemanse et al., 2020: 2). We do not have to look far to find people making use of iconicity during their everyday interactions. For example, a hearing Siwu speaker produces the spoken ideophone *shû* *shû* while moving his hands upwards quickly to show that flames will flare upwards quickly after he sets two piles of gunpowder on fire (Dingemanse, 2013: 158). A hearing Ngaanyatjarra speaker using *mara yurriku* (‘sign language’ or ‘signing’, *lit.* ‘moving the hands’) traces the orthographic letters *AS* in the air while speaking to refer to the town of Alice Springs (Ellis et al., 2019: 105). A deaf signer of Norwegian Sign Language places her palms together on one side of her face while tilting her head and closing her eyes to show a boy falling asleep for the night (Ferrara and Halvorsen, 2017: 385). While conversing with his deafblind aunt, a deafblind signer of Bay Islands Sign Language guides her hands to his face, so that she can feel him produce the mimetic head movement and facial mannerism that has long been the name sign of her youngest brother (Ali, 2020). Even without moving their hands or body, hearing English speakers make frequent use of iconicity, as evidenced by the prevalence of words such as *sniff, murky,* and *buzzing*, each selectively profiling the different sensorial qualities of various perceptual experiences (Winter et al., 2017).

Researchers from a range of disciplines have collectively demonstrated that iconicity is fundamental to human communication and language use (Peirce, 1931-1958; Jakobson, 1965; see Mandel, 1977; Haiman, 1980; Parmentier, 1994; Wilcox, 2004; Cuxac and Sallandre, 2007; Perniss et al., 2010 and many others). However, defining and operationalising construals of iconicity across different interactions (e.g., deaf, deafblind, hearing), modes of communication (e.g., manual signs, speech, writing), and languages (e.g., English, Japanese, Auslan) remain a slippery matter (Perniss et al., 2020). Researchers using experimental approaches have primarily viewed iconicity as perceptual resemblances construed in at least three different ways: (i) as a discrete property that is present or absent; (ii) as semiotic relations that come in kinds; and (iii) as scalar substance that comes in degrees (Dingemanse et al., 2020). As Dingemanse et al. (2020) explain, each construal helps to reveal different aspects of how perceptual resemblances manifest in language use and interaction, yet each one has limitations.

For example, when iconicity is operationalised as a discrete, categorical property (i.e., present or absent) or as a binary (e.g., strong vs. weak, iconic vs. arbitrary), it often falls apart when applied to real life language use in situated contexts (Blasi et al., 2016). When more fine-grained analyses of the dynamic, semiotic relations occurring within situated contexts are undertaken, it is often not clear if the resulting descriptive complexity is useful for understanding how people use or learn language in a principled way (Esposito, 1979). When iconicity is operationalised as a scalar substance perceived in varying degrees, results suggest that perceived iconicity is best explained by people’s subjective experiences with their languages and modes of communication, rather than any objectively defined quality such as transparency, thus problematising the comparison of iconicity ratings elicited from signers and nonsigners (Occhino et al., 2017). There is not yet a unified construal of iconicity that addresses these limitations.

The situation is complicated by various hegemonic biases that have contributed to the marginalisation or pathologisation of different language and communication phenomena across the language sciences (Sicoli, 2014; Dingemanse, 2017; see also Goodwin, 1995). This marginalisation includes aspects of how iconicity is created and used during interactions between people who are deaf, deafblind, and/or disabled; between people who have sensorial asymmetries; and/or between people who have simply not been the focus of Western science in general (see Kusters et al., 2017; Di Paolo et al., 2018; Braithwaite, 2020). It also includes aspects of iconicity beyond material perceptual resemblances, such as diagrammatic iconicity and metaphorical iconicity (Haiman, 1985; Hiraga, 1994; Müller and Cienki, 2008). Yet if we are to strive for a comprehensive understanding of language and communication, it is necessary to remedy these biases and seek continuity across the various manifestations of iconicity evidenced in our interactions, as well as our methods for investigating them (Perniss and Vigliocco, 2014; Dingemanse et al., 2020). Only then can we do justice to human social complexity in our efforts to understand how languaging works and why it differs.

In the following sections, we outline two main issues with how iconicity has been defined and operationalised in the language and communication sciences. The first issue relates to the prominence of *form* in analyses of iconicity, and how iconicity is typically framed in terms of bounded language modalities (‘spoken language’, ‘signed language’, ‘verbal modality’, ‘gestural modality’), modes of communication (‘speech’, ‘sign’, ‘gesture’) and/or small, single units (‘words’, ‘signs’). The second issue relates to the prominence of *perceptual resemblances* in analyses of iconicity, without concurrently considering other kinds of resemblances, such as resemblances of *relation* and *association*. We offer some correctives by drawing on semiotic approaches to language and communication, especially the model of language use as signalled through describing, indicating, and/or depicting (Clark, 1996). Our aim is to illuminate the multidimensionality of iconicity in co-present interactions, thereby encouraging more unified progress in our understanding of how it works and why we use it. This builds on our earlier proposal for analysing how these different methods of signalling are combined in multimodal language use (Ferrara and Hodge, 2018).

We then apply this framework to a range of interactions documented in the literature to interrogate more closely how and why different aspects of these interactions look, feel, sound, or otherwise resemble what they mean. We consider how iconicity is integrated with other semiotics and bodily articulations within composite utterances (Enfield, 2009). We also consider how iconicity is used in terms of both referential and non-referential functions (Silverstein, 1976). In this way, we outline a semiotic construal of iconicity that can be operationalised across different interactions, modes of communication, and units of analysis. This construal aligns with others who broadly recognise iconicity as multimodal, polysemiotic, and plurifunctional (e.g., Nöth, 1999; Kendon, 2004; Mittelberg and Waugh, 2009; Green, 2014; Perniss and Vigliocco, 2014; Kok et al., 2016; Wilcox and Occhino, 2016; Iriskhanova and Cienki, 2018; Puupponen, 2019; Bressem, 2020; Murgiano et al., 2020). Finally, we discuss some implications for the language and communication sciences, and explain how this approach guides us towards a theory of biosemiotics.

## Issues With Defining and Operationalising Iconicity

### The Prominence of Form in Analysing Iconicity

There are two main issues with how iconicity has been defined and operationalised. The first issue is the prominence of *form* in driving investigations of iconicity, which results from the traditional paradigm to ‘focus on the means at the expense of the content’ (Wacewicz and Żywiczyński, 2015: 37). Most studies have focused on iconic forms relating to specific modes of communication and/or single units. For example, spoken language researchers have investigated lexical spoken words such as ideophones, including onomatopoeia, and other types of sound symbolism, such as modifications to word length signifying smallness or lightness (e.g., Diffloth, 1994; Nuckolls, 1999; Dingemanse, 2012). Signed language researchers have analysed the iconic aspects of conventionalised manual signs, which are usually considered the closest equivalent to lexical words in spoken and written languages (e.g., DeMatteo, 1977; Taub, 2001; Padden et al., 2013). Others have analysed the iconicity of aspectual modifications, verb agreement, constructions and the meaningful use of space more generally (e.g., Engberg-Pedersen, 1993; Emmorey et al., 2000; Gray, 2013; Hou, 2018).

There is an extensive literature on the iconic dimensions of manual gestures with and without speech (e.g., McNeill, 1985; Goldin-Meadow, 2003; Kendon, 2004; Streeck, 2008; Müller, 2014). The close relationship between iconic manual gestures and spoken forms has been emphasised with respect to synchronous timing, semantic categories, and how language and speech influence the use of manual gestures and vice versa (e.g., Kita and Özyürek, 2007; Özyürek et al., 2008). Signed language researchers have also investigated iconicity in less conventionalized forms, such as tokens of partly or fully improvised manual signs that depict the shape and/or movement of an object (‘classifier signs’, ‘depicting signs’) and visible bodily enactments (‘personal transfers’, ‘constructed action’, ‘quotation’, ‘role shift’; e.g., Cuxac, 1999; Liddell, 2003; Cormier et al., 2015; Davidson, 2015).

As scientific understandings of iconicity across languages and modalities have developed, so has interest in cross-linguistic and cross-modal comparisons. Specific iconic forms, such as spoken ideophones, have been compared across languages (e.g., Kilian-Hatz, 2001; Dingemanse, 2012). Various iconic forms have also been compared across languages and modalities, including comparisons of the manual depicting actions used by signers and speakers of different languages (e.g., Schembri et al., 2005; Cormier et al., 2012) and comparisons of lexical iconicity across signed and spoken languages (e.g., Hwang et al., 2016; Perlman et al., 2018). Researchers have also investigated how iconicity manifests more generally in the lexicon of spoken and signed languages (e.g., Waugh, 1994; Padden et al., 2013). Others have proposed hypotheses for cross-linguistic, cross-modal comparison of phenomena such as aspectual modifications, depicting constructions, ideophones, constructed actions, and mouth actions (e.g., Bergman and Dahl, 1994; Ajello et al., 2001; Pizzuto et al., 2008; Padden et al., 2013; Sallandre et al., 2016; Ferrara and Halvorsen, 2017; Lu and Goldin-Meadow, 2018; Akita, 2019).

A key point of interest for many is the suggestion that the visual and spatial affordances of signed languages facilitate different and potentially greater use of iconicity compared to spoken languages (see Perlman, 2017, for an overview). This idea stems from the observed homeomorphism (i.e., topological isomorphism) between the multidimensional world around us and the multidimensional nature of signed interactions, which has resulted in strong claims about signed languages being ‘more iconic’ than spoken languages (e.g., Klima and Bellugi, 1979; Taub, 2001; Pietrandrea, 2002). Yet empirical investigations of iconicity in spoken, signed, and even nonhuman primate communication have shown that iconicity is abundant, motivated, and systematic, regardless of whether it is spoken, signed, or vocalised (see Perniss et al., 2010; Dingemanse et al., 2015; Perlman, 2017). Instead, it may be that different modes of communication are shaped by different affordances, so that iconicity manifests across interactions and languages in patterned ways (Perniss and Vigliocco, 2014; Dingemanse, 2019; see also Caselli et al., 2022). For example, manual actions may be best suited for depicting what something looks or feels like, or how it is handled, whereas vocalisations may be best suited for depicting how things sound or smell (see e.g., Padden et al., 2013; Hou, 2018; Majid, 2020; Keränen, 2021).

A further key point is the suggestion that iconicity motivates grammar and is therefore an explanatory principle for the emergence of language (see Haiman, 2008; Meir et al., 2013). However, when different types of iconicity are teased apart and investigated, it is sometimes found to be not the only motivating factor, with some patterns best explained by other principles such as frequency of use (see Haspelmath, 2008). It is also not always clear that like is being compared with like. For example, Perlman et al. (2018) compared iconicity ratings of various lexical forms evidenced in American Sign Language (ASL), British Sign Language (BSL), English, and Spanish. They used a broad and impressive range of semantic categories in their analysis, including a category ‘other grammatical words’, such as the second person singular pronoun form used in each language (PT:PRO2SG,^1^ *you* and *tú*). This category in ASL and BSL was rated significantly more iconic than for English and Spanish (see Perlman et al., 2018: 11). However, these forms are primarily indexical, so it was indexicality that was tested across these forms, not iconicity. Furthermore, English and Spanish speakers also often use visible finger-pointing actions in conjunction with spoken indexical forms such as *you* or *tú*, and ASL and BSL signers often use such pointing forms in conjunction with mouthings of forms such as *you*. Thus, while it is defensible that ‘iconicity ratings really do measure iconicity’ (Winter and Perlman, 2021: 8), this example demonstrates that like is not always being compared with like during cross-linguistic and cross-modal comparisons, and that there is a risk that indexicality is conflated with iconicity. We propose that deeper interrogation of iconicity—as an interpretation, an effect and an explanatory principle—is warranted.

### The Prominence of Iconicity as Perceptual Resemblances

The second issue with how iconicity has been defined and operationalised relates to how iconicity as material *perceptual* resemblances has been prioritised, without also considering resemblances of *relation* and/or *association*. For example, Perniss and Vigliocco (2014: 2) define iconicity as ‘any resemblance between certain properties of linguistic/communicative form and certain sensori-motor and/or affective properties of corresponding referents’. However, semiotician Charles S. Peirce (1839–1914) differentiated at least three dimensions of iconicity: (1) imagistic iconicity; (2) diagrammatic iconicity; and (3) metaphoric iconicity (CP 2.277; see also Hiraga, 1994; Nöth, 1999). The resemblances provoked through these three types of iconicity are often drawn from different sources, and they are not mutually exclusive. Imagistic iconicity is resemblance in quality, while diagrammatic iconicity is resemblance in relations or structure, and metaphoric iconicity is resemblance by association (Hiraga, 1994; Radwańska-Williams, 1994). The next paragraphs provide examples of these three types of iconicity as defined here.

Imagistic iconicity is about how given forms look, sound, feel, or otherwise materially and selectively resemble what they mean. For example, the first photographic self-portrait ever taken (c.1839) renders the man who was Robert Cornelius into a quarter plate daguerreotype (Carbon, 2017); the spoken Japanese ideophone *don don* echoes a loud drumming or thumping sound (Kakehi et al., 2011); and the manual ASL (American Sign Language) sign TREE^2^ visibly depicts the trunk and branches of a living tree (Klima and Bellugi, 1979). Examples such as *don don* and TREE align closely with the definition of linguistic iconicity offered by Perniss and Vigliocco (2014) and widely adopted by others, but there are still at least two other types of iconicity that must be considered.

Diagrammatic iconicity is about how the systematic arrangement of different forms somehow mirrors the relationship between the things they reference. For example, the famous map of the London Underground mirrors the relations between different tube lines and stops along each line (Atã et al., 2014); the sequence of conjugated verbs in the phrase *veni, vidi*, *vici* attributed to Julius Caesar mirrors the order in which these events occurred (Jakobson, 1965); and the spatially motivated Auslan utterance POLICE CATCH THIEF mirrors both the spatial and agentive relations between policeman and thief (Johnston, 1996: 72). Diagrams do not perceptually resemble their object; they are better understood as a generality or schema (Stjernfelt, 2019). As such, diagrammatic iconicity manifests through the relations inferred by intentionally combining multiple forms, referents, and/or units.

Metaphoric iconicity, which Peirce mentions only briefly in his work, represents ‘a parallelism in something else’ and instantiates a triadic relationship between a sign, an object, and that ‘something else’ (Hiraga, 1994: 7). This relationship is ‘beheld as an image in the mind’s eye’ (Radwańska-Williams, 1994: 23). In other words, metaphor is what happens when we express one idea, experience, or semantic domain in terms of another (see Lakoff and Johnson, 1980). Metaphors often manifest both imagistic and diagrammatic iconicity. For example, the oil painting *Judith Slaying Holofernes* (c.1620) resembles both the heroism of the biblical Judith slaying her enemy Holofernes, and the artist Artemisia Gentileschi avenging her rapist Agostino Tassi (Gotthardt, 2018); the ASL signs ANALYSE,^3^ SURFACE,^4^ and DEEP^5^ all draw on the conceptual metaphor ANALYSIS IS DIGGING, relating depth of knowledge with physically digging into the ground to reveal objects (Taub, 2001); and the English expression *‘my love is a rose’* signifies its object (*my love*) via a parallelism with something else (*a rose*; Hiraga, 1994).

While imagistic iconicity often manifests in single forms (e.g., words, signs), diagrammatic and metaphorical iconicity typically require larger sequences or communicative moves for their intended interpretation (e.g., clauses, composite utterances). In spoken language interactions at least, diagrammatic iconicity often relies on relationships between single forms composed within constructions, and metaphorical iconicity often relies on multi-form utterances (see, e.g., Hiraga, 1994, for a discussion of grammatical and conventional metaphor). Of course, we now accept that metaphorical iconicity in signed languages and co-speech gestures may be expressed in both single and multi-form constructions (Taub, 2001; Mittelberg, 2008). However, the heavy focus on analysing single forms or units may partially account for the inattention to diagrammatic and metaphoric iconicity during investigations of imagistic iconicity.

### Imperatives for Defining and Operationalising Iconicity

Regardless how iconicity is defined and operationalised, one imperative is to recognise the semiotic diversity of human languaging by considering the range of bodily actions that people intentionally and jointly coordinate during their interactions, no matter how conventionalised these actions are or how they are articulated (Goodwin, 1986; Bavelas, 1990; Johnston, 1996; Kendon, 2004). Another is to recognise the multilingual and multimodal repertoires that different people and communities develop and draw upon in different contexts and for different (socio)linguistic and cultural reasons (Silverstein, 1976; Halliday and Hasan, 1989; Busch, 2012; Kusters et al., 2017). This entails moving beyond concepts of languages as bounded modalities to concepts of languaging as making use of the semiotic repertoires available within specific interactions and spatiotemporal contexts (see Kusters et al., 2017).

It is the semiotic intent which at least partly triggers how an utterance manifests (see Cuxac and Sallandre, 2007). As the examples described above demonstrate: if it is intended as meaningful within an interaction, it must be considered. The conceptual tools used for such investigations must also be ‘modality-agnostic’ (Dingemanse, 2019: 25). This is the aim of comparative semiotics, whereby various aspects of language and communication are compared across interactions, modes of communication, and languages (Kendon, 2008, 2014). In doing so, we can move beyond essentialist dualisms of ‘signed vs. spoken languages’, ‘aural-oral vs. visual-gestural modalities’, ‘iconicity vs. arbitrariness’, and ‘convention vs. improvisation’ to build a richer understanding of all our commonalities and differences, including how and why these emerge. In the next section, we draw on the model of language use as signalled via describing, indicating, and/or depicting (Clark, 1996) to build on these imperatives for a modality-agnostic, comparative semiotics of iconicity.

## Language Use as Signalled via Describing, Indicating, and Depicting

### Making Language Theory More Inclusive

In Ferrara and Hodge (2018), we argued that a theory of language must account for the wide range of communicative practices used across the world, beyond speaking and writing. In order to make language theory more inclusive, we expanded on the proposal by Clark (1996) that language use is actioned via three methods of signalling, which he termed *describing-as*, *indicating*, and *demonstration*. This builds on Peirce’s second trichotomy (*symbols*, *indices*, and *icons*), which was first applied to linguistics by Jakobson in his appeal for linguists to consider more the dynamic nature of signs (broadly defined) and the many relations between them (Jakobson, 1965; see Nöth, 1999). We reframed these three methods as *describing*, *indicating*, and *depicting* to correspond with more recent analyses of signed and spoken language interactions (e.g., Liddell, 2003; Dingemanse, 2013; Clark, 2016).

The central idea is that during our interactions, we use these three methods of signalling in varying degrees to create words, signs, grammatical constructions, composite utterances, and so on. Our communicative moves, such as composite utterances, involve combining different forms created with these three methods of signalling (Clark, 1996; see also Johnston, 2013; Puupponen, 2019; Cooperrider et al., 2021; Capirci et al., 2022). This approach aligns closely with other approaches developed through the analysis of signed language use, such as the Semiological Approach and Cognitive Linguistics frameworks, and comparative semiotics more generally (e.g., Cuxac, 1999; Cuxac and Sallandre, 2007; Ferrara, 2012; Wilcox and Occhino, 2016; Jantunen, 2017; see Garcia and Sallandre, 2020 and Capirci et al., 2022, for overviews). The three methods of signalling are summarised below in the rearranged order of *indicating, depicting*, and *describing* to more closely reflect the complex ontogeny of human communication (see Bruner, 1983; Tomasello, 1995; Diessel, 2006).

### Signalling by Indicating

*Indicating* refers to how people index and anchor communicative intent to a particular time and place. In Peircean terms, it is the method of signalling with indices. As such, indicating depends on grounded contexts for accurate interpretation. Indicating combines conventional and non-conventional properties, and primarily functions to focus another’s attention on specific referents in the discourse and/or situated context. Token finger-pointing actions and spoken indexical symbols such as English *this* or *she* are examples of indicating: the form is conventionalised, but accurate interpretation depends on recognising which referent one’s attention is being anchored to. As these tokens are conventionalised, they also describe (see the section Signalling by Describing). Clark (2003) further differentiated indicating as *directing-to*, which involves directing attention to specific referents, and *placing-for*, which involves placing objects meaningfully within an interactant’s field of attention. For example, when a person extends their arm to direct attention to their own car among many others in the car park, they are indicating by *directing-to*. When a person intentionally places a card on a table during a card game, they are indicating by *placing-for*. Thus, *placing-for* can be continuous and always involves an element of *directing-to*, whereas *directing-to* is transitory and does not necessarily involve *placing-for*. Both can manifest diagrammatic iconicity by creating relations between different referents (see also Wilcox and Occhino, 2016). Signed interactions often incorporate both kinds of indicating through visible or tactile pointing, tracing, and/or placement of signs (Edwards, 2015; Wilcox and Occhino, 2016; Martínez and Wilcox, 2019; Beukeleers, 2020).

### Signalling by Depicting

*Depicting* refers to how people use resemblances of quality, relation, and/or association to show meaning. In Peircean terms, it is the method of signalling with icons. Most of the literature on depicting has focused on the use of imagistic iconicity to demonstrate what something looks, sounds, feels, smells or tastes like, so that we ‘imagine what it is like to see the thing depicted’ (Dingemanse, 2015: 950). Tokens of spoken ideophones, representational co-speech gestures, and bodily enactments that reconstruct what someone did or said are all examples of depicting in spoken language interactions (e.g., Kunene, 2001; Heath, 2002; Park, 2009). These forms can vary in degree of conventionalisation and/or their use of indicating by *directing-to* and *placing-for*. As such, they can be understood as compositions of depicting, describing, and/or indicating.

For example, Clark (2016) outlines a detailed typology of depicting in communication, focusing on how depicting can be signalled within speech utterances that also describe (see also Hsu et al., 2021). Among signed language researchers, there has been much discussion about depicting via iconic lexical signs (e.g., Frishberg, 1975; Mandel, 1977; Ferrara and Halvorsen, 2017; Lepic and Padden, 2017), partly conventionalised depicting signs (e.g., Supalla, 1982; Cuxac, 1999; Liddell, 2003) and bodily re-enactments of actions and utterances (e.g., Metzger, 1995; Cuxac, 1999; Cormier et al., 2015; Sallandre et al., 2016; Jantunen, 2017). These often also involve indicating and/or describing.

As explained above, the concept of depicting has typically been defined as manifesting imagistic iconicity. However, if we consider depicting more broadly as the creation of resemblances, we must also include diagrammatic and metaphorical iconicity in our definition. For example, some spoken ideophones exhibit ‘quantity iconicity’ in addition to imagistic iconicity, so that more form equates to more meaning (Hiraga, 1994; Bressem, 2020; although *cf.* Haspelmath, 2008, who argues that frequency of use is the only explanation necessary for quantity iconicity). Some co-speech gestures exhibit metaphorical iconicity, such as when a cupped hand refers to an abstract entity (Mittelberg, 2008; Iriskhanova and Cienki, 2018). An instantiation of quantity iconicity, such as the Auslan sign GIVE^6^ meaningfully directed to a referent located in space and produced with multiple iterations to signal plurality, can be understood as concurrently manifesting imagistic and diagrammatic iconicity, while a CUPPED HAND gesture manifests imagistic and metaphorical iconicity.

The definition of depicting can therefore be recalibrated to more broadly encompass imagistic, diagrammatic, and/or metaphorical iconicity. This enables us to mitigate the hyper-focus on imagistic iconicity, while also respecting the meaning-making that emerges through other kinds of iconicity (see the section Imperatives for Defining and Operationalising Iconicity). Thus, diagrammatic iconicity, which includes relational resemblances such as temporally isomorphic word order patterns and referential use of the signing space, and metaphoric iconicity, which can be identified in the single form of some signs and co-speech gestures as well as in more complex constructions, are analysed as depicting. We are then forced to consider more deeply how iconicity manifests within and across composite utterances, concretely through to schematically, and through different compositions of signalling in varying degrees and complexities.

### Signalling by Describing

*Describing* refers to how people use agreed-upon forms to prompt more contingently stable meanings. In Peircean terms, it is the method of signalling with symbols. Describing is primarily interpreted and understood through conventions across communities of use. For example, the words *jour* and *nuit* are two examples of conventionalised symbols used by French speakers to refer to what English speakers know as *day* and *night* (Jakobson, 1965). The emblematic manual gestures MANO A BORSA (‘purse hand’) and MANI GIUNTE (‘praying hands’) used in Southern Italy to either express disbelief or make an entreaty are acts of describing, as are the conventionalised rising intonation contours that English speakers use to signal they are asking a question (Bolinger, 1983; Kendon, 1995). There are also many other regularities of language use that conventionalise and may therefore describe, such as specific word order patterns for disambiguating who did what to whom, and the agentive case marking patterns of Tibeto-Burman languages used to disambiguate the agent from other referents (Silverstein, 1976; Lapolla, 1995). Conventionalised symbols used to solve problems of understanding, such as the many forms of *huh?* that have evolved to initiate conversational repair, also describe (Schegloff et al., 1977; Dingemanse et al., 2013).

Describing also incorporates what de Saussure and others have observed as ‘arbitrary’ forms without any obviously motivated links between the given forms and their intended meanings. Yet it is important to recognise that arbitrariness is not an inherent or defining property of describing (*cf.* Hockett, 1960). Rather, arbitrariness is a consequence of our aptitude for abstracting *x* from multiple instantiations *i, ii, and iii*, so that subsequent instantiations are understood as *x* even when decontextualised (see Parmentier, 1994; Bybee, 2007; Cuxac and Sallandre, 2007). The ability to create and interpret symbols, and therefore to signal by describing, depends on an interpretant first experiencing a triadic relation between themself, the referent and its associated form (CP 2.298; see also Radwańska-Williams, 1994; Mittelberg, 2019). It is through conventionalisation that descriptions can be arbitrary and discrete (Dingemanse, 2015). Thus, strategies for describing tend to evolve comparably late in the ontogeny of human semiosis, occurring only after one experiences such triadic relations in the first instance—relations that are typically initiated and interpreted through acts of indicating and/or depicting, but also scaffolded by the development of turn-taking and repair practices (see Kelly, 2006; Clark, 2020). As Peirce noted, ‘Symbols grow. They come into being by development out of other signs, particularly from icons, or from mixed signs partaking of the nature of icons and symbols’ (CP 2.302). For example, some lexical signs can be analysed as both icons and symbols, and sometimes also indices (see the section Iconicity as Signalled by Depicting, Indicating, and/or Describing).

### Signalling by Indicating, Depicting, and Describing

These three methods of signalling—indicating, depicting, and/or describing—facilitate potentially infinite possibilities for meaning-making and building shared understanding through interaction. The examples provided in the previous sections illustrate the importance of recognising that each method is typically used in combination with other methods to create composite signals. As Peirce observed, ‘a single sign may have iconic, indexical, and symbolic properties’ (CP 4.447). As we will show in the section ‘Comparing Iconicity Across Interactions’, it is rare to observe a languaging form resulting from ‘pure’ indicating, ‘pure’ depicting, or ‘pure’ describing (Ferrara and Hodge, 2018; see also Capirci et al., 2022). For example, finger-pointing actions used to direct attention to real or imagined referents are widely regarded as the paragon of indicating in co-present communication (Tomasello, 2003; Cooperrider et al., 2014). Yet while these actions primarily indicate, they also describe, because the form of indicating may be both culturally and semantically specific (Wilkins, 2003; see also Johnston, 2013). It is simply that in cases of finger-pointing to indicate, the indexical qualities of the pointing actions are more prominent than other co-existing symbolic qualities (see also Johnston and Schembri, 1999; Cooperrider et al., 2021).

This principle of polysemiosis is often overlooked, yet it has significant implications for how iconicity is defined and operationalised across interactions, modes of communication, and languages.^7^ As Jakobson recognised early on, ‘the iconic and indexical constituents of verbal symbols have too often remained underestimated or even disregarded; on the other hand, the predominantly symbolic character of language and its subsequent cardinal difference from the other, chiefly indexical or iconic, sets of signs likewise await due consideration in modern linguistic methodology’ (Jakobson, 1965: 36). Indeed, Peirce concluded that ‘the most perfect of signs are those in which the iconic, indicative, and symbolic characters are blended as equally as possible’ (CP 4.448). Regardless which framework is used, when we talk about iconicity in language and communication, we are not just talking about depicting; we are talking about depicting, indicating, and/or describing combined in different ways. To emphasise iconicity as involving depicting alone, while ignoring any indicating and/or describing signals, is to reinforce a category error that has significant implications for how we investigate and compare iconicity across interactions.

## Iconicity as Signalled by Depicting, Indicating, and/or Describing

### Recognising Iconicity as Multimodal and Polysemiotic

**Figure 1:**
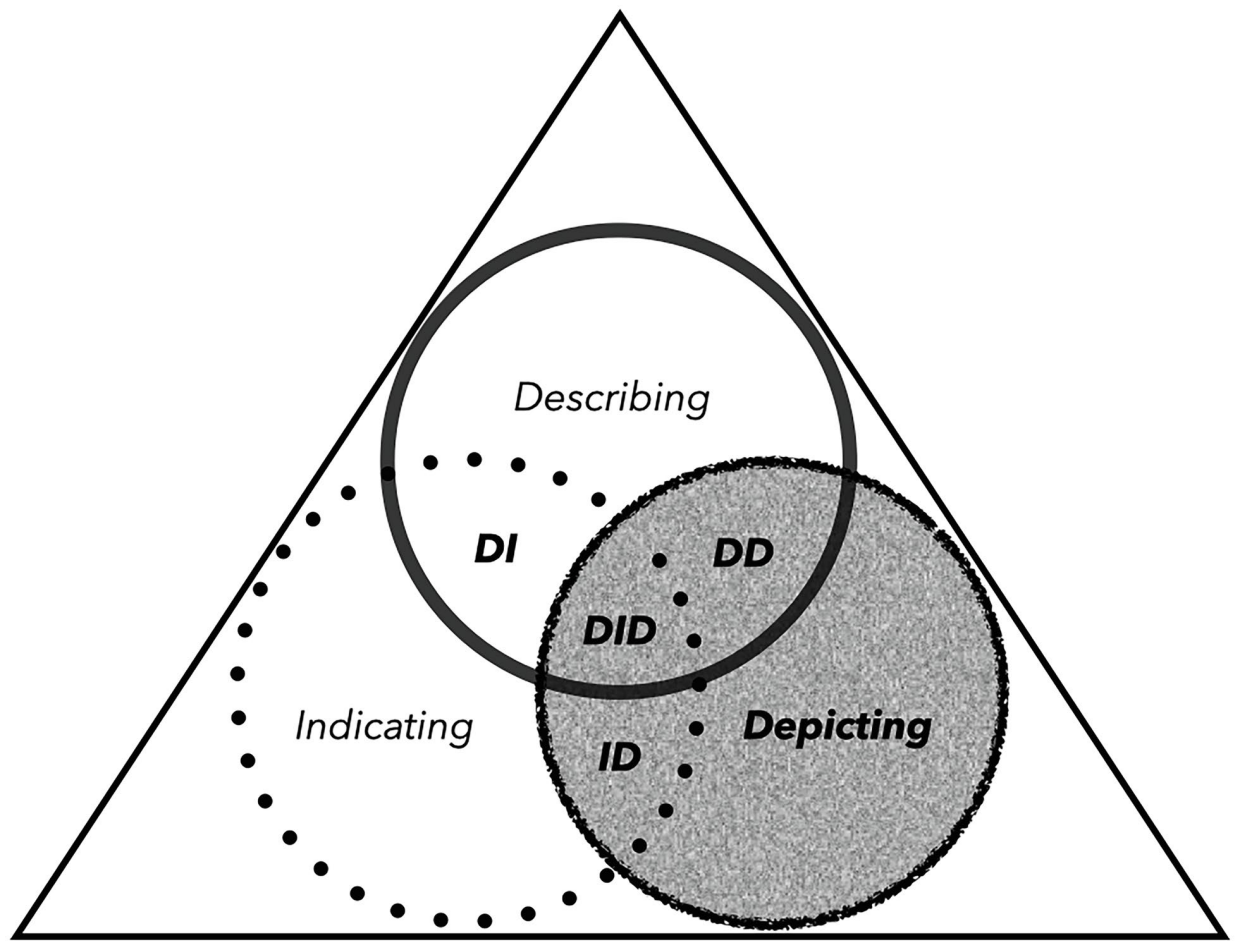
Iconicity (shaded grey) as depicting, indicating, and/or describing.

So far we have considered how iconicity is multimodal and polysemiotic. In this section, we consider how these two dimensions of iconicity may be reconceptualised in language theory and operationalised in analytical practice. We want to demonstrate that iconicity minimally involves depicting, but signalling solely by depicting is rare. Iconicity usually also involves indicating and/or describing, and often with more than one bodily articulator and/or situated semiotic resource, such as a shop counter. Figure 1 illustrates the three methods of signalling as circles enclosed within a Peircean triangle. These circles do not represent bounded semiotic categories; they are intended to conceptually represent the potentialities of iconicity in terms of signalling through depicting, indicating, and/or describing. It is the triangle itself that can potentially represent a token form or aspects of an utterance (see also Puupponen, 2019; Capirci et al., 2022). In this way, iconicity can be reconceptualised as anything falling into the shaded grey areas. At least four polysemiotic manifestations of iconicity are possible: (i) depicting and indicating; (ii) depicting and describing; (iii) depicting, indicating, and describing; and (iv) depicting alone.

We now revisit examples of iconicity evidenced in a range of interactions and utterances documented in the literature, and consider how this reconceptualisation of iconicity can be operationalised in linguistic analysis. Some of the examples were originally analysed as iconic forms, while others were specifically chosen to redress bias in the field and further illustrate the framework proposed here. In each example, we consider how iconicity is signalled via depicting, indicating, and/or describing during the utterance, and whether these resemblances are imagistic, diagrammatic, and/or metaphorical. This approach offers liberation from the issues described in the sections ‘The Prominence of Form in Analysing Iconicity’ and ‘The Prominence of Iconicity as Perceptual Resemblances’, while upholding the imperatives outlined in the section ‘Imperatives for Defining and Operationalising Iconicity.’ It also highlights the composite multimodal and polysemiotic signalling within utterances as continuous and contingent processes, in addition to interpreting the token forms in each utterance as bounded, meaningful units.

In each example, we ask two questions: (i) how does the interaction signal depicting, indicating, and/or describing? (ii) how does the interaction manifest imagistic, diagrammatic, and/or metaphorical iconicity? Each figure is annotated with dotted lines (indicating), soft lines (depicting), and/or sharp lines (describing). These lines are intended to capture the prominence and co-occurrence of indicating, depicting and/or describing as the utterance unfolds in real time.^8^ The number of lines represents the number of bodily articulators involved in signalling each method at a given moment, which are also labelled on the right hand side of the figure. The imagistic resemblances within each example are enclosed within a green dotted box. The diagrammatic resemblances are enclosed within a green dashed box. Metaphoric resemblances are enclosed within a green lined box. Our analysis demonstrates that while these interactions each manifest iconicity, each manifestation is iconic in its own way.

### Analysing Iconicity in Interactions

**Figure 2:**
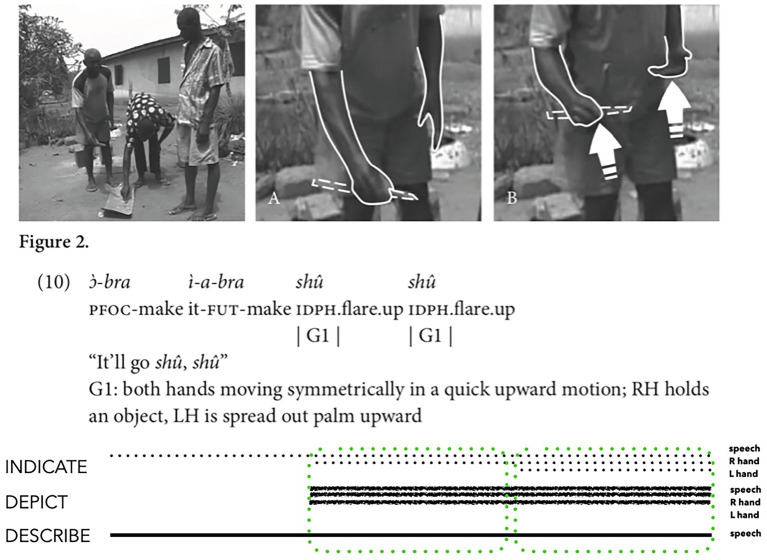
Composite utterance produced by a hearing speaker of Siwu (adapted from Dingemanse, 2013: 158 and reproduced with permission from the author and John Benjamins Publishing Company, Amsterdam/Philadelphia, https://benjamins.com/catalog/gest).

Dingemanse (2013: 158) analysed how hearing Siwu speakers coordinate spoken ideophones with manual depicting actions, documenting an interaction where one speaker produced the spoken ideophone *shû shû* while moving his hands upwards quickly to show how flames will flare upwards after he sets two piles of gunpowder on fire. In this instance, the speaker uses a speech and manual action ensemble to depict, indicate, and describe the look and sound of the flames (Figure 2, Image B). Three bodily articulations (speech and two hands) depict the audible and visible qualities of flames quickly flaring upwards. The sound quality and syllabic repetition of the spoken form *shû shû* depicts the audible qualities of these flames, while the upturned handshape, upward direction and repeated movement of the two-handed manual action depict the visible qualities of these flames. The initially low placement of the man’s two hands indicates the gunpowder by *placing-for*, while the upward movement of the hands indicates by *directing-to*. The spoken form *shû shû* is a conventional ideophone for these speakers; hence, this form also describes. Altogether, the ensemble signals imagistic iconicity via depicting (speech and two hands), indicating (hands only) and describing (speech only). The prosodic aspects of the speech may also signal depicting, indicating, and/or describing, but we do not have access to this detail here.

**Figure 3:**
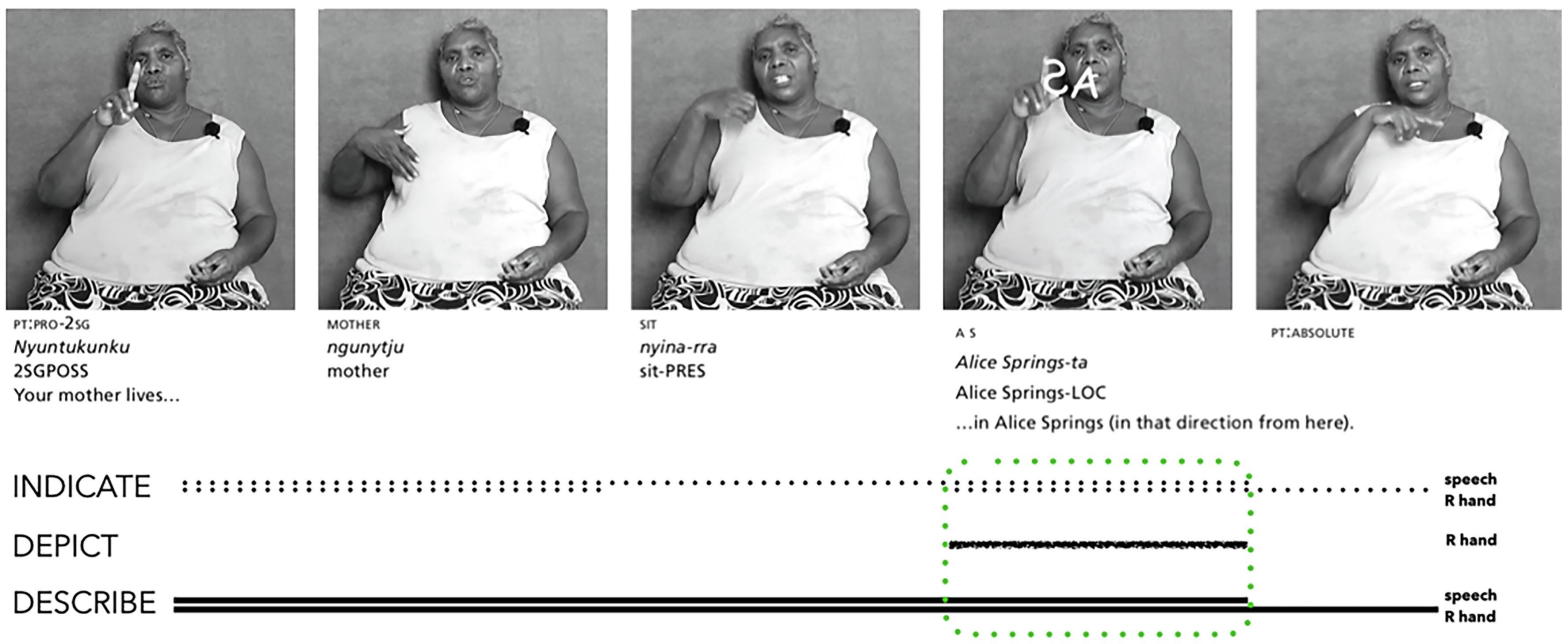
Composite utterance produced by a hearing Ngaanyatjarra/Ngaanyatjarra speaker (adapted from Ellis et al., 2019: 105–106 and reproduced with permission from the authors).

Ellis et al. (2019) describe the wide range of signing practices used by Aboriginal communities of the Ngaanyatjarra Lands in the Western Desert of Australia. These include repertoires of conventionalised manual signs that may be used with or without speech; and air writing, whereby one traces the letters of a word on one’s arm or leg, or in the sand or air. The first author, Elizabeth Marrkilyi Ellis, is a highly respected Ngaanyatjarra/Ngaatjatjarra speaker who is well versed in these signing practices. Figure 3 illustrates how she combined air writing, manual signs, and speech to identify where an interactant’s mother was living (Ellis et al., 2019: 105). In this composite utterance, Ellis creates an air writing and speech ensemble that depicts, indicates, and describes the place name *Alice Springs*. She coordinates two bodily articulations (one-handed actions and speech) to trace the outline of the letters *AS* in the air while speaking the forms *Alice Springs-ta* (*lit.* ‘*Alice Springs in that direction from here*). Her manual tracing actions prompt imagistic iconicity by resembling the conventional letters A and S, which emerges by both *directing-to* and *placing-for* these letter shapes in the air. The co-occurring English speech describes the location using the conventionalized English place name, and the Ngaanyatjarra speech describes and indicates the location using the conventionalized Ngaanyatjarra locative form. These speech forms also visibly index these English and Ngaanyatjarra words for people who cannot hear. Altogether, the imagistic iconicity of the Alice Springs ensemble is signalled by depicting (one hand), indicating (speech and hand) and describing (speech and hand).

**Figure 4:**
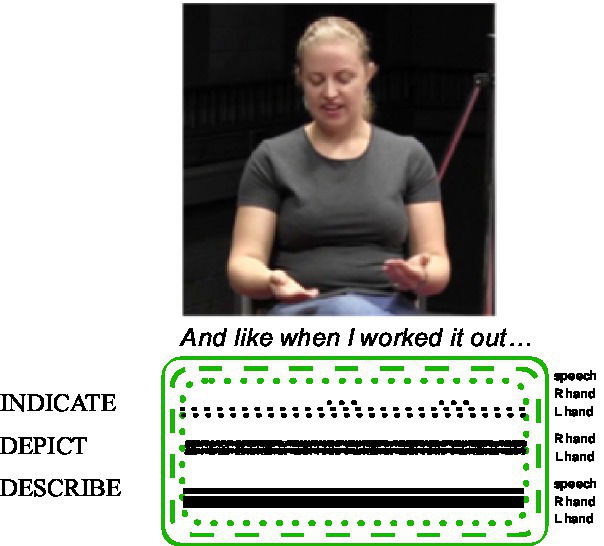
Composite utterance produced by an Australian English speaker (adapted from Ferrara and Hodge, 2018: 11).

Ferrara and Hodge (2018: 11) analyse a composite utterance produced by a hearing speaker of Australian English who is comparing the price of plane tickets from two different airlines (see Figure 4). The speaker says *‘When I worked it out’*, while moving her left hand slightly upwards and downwards, as her right hand remains stable. In this composite utterance, the speech and manual action ensemble depict, indicate, and describe the two ticket options by using the metaphor COMPARISONS ARE SCALES. The speaker uses her two hands to depict the opposing surfaces of a scale being weighed, or two calculations being compared, so their distal relationship in space exhibits diagrammatic iconicity. As this metaphor is conventionally used to express CONTRAST for English speakers, these manual actions also describe (see Hinnell, 2019). The placement of the hands in space in relation to each other, while the speaker directs her eye gaze to them, are acts of indicating, as are the conventional spoken English words *I* and *it*. The imagistic, diagrammatic, and metaphoric iconicity manifested in this ensemble are signalled by depicting (two hands), indicating (speech and eye gaze) and describing (speech and two hands). The prosodic aspects of the speech may also signal depicting, indicating, and/or describing, but we do not have access to this detail here.

**Figure 5:**
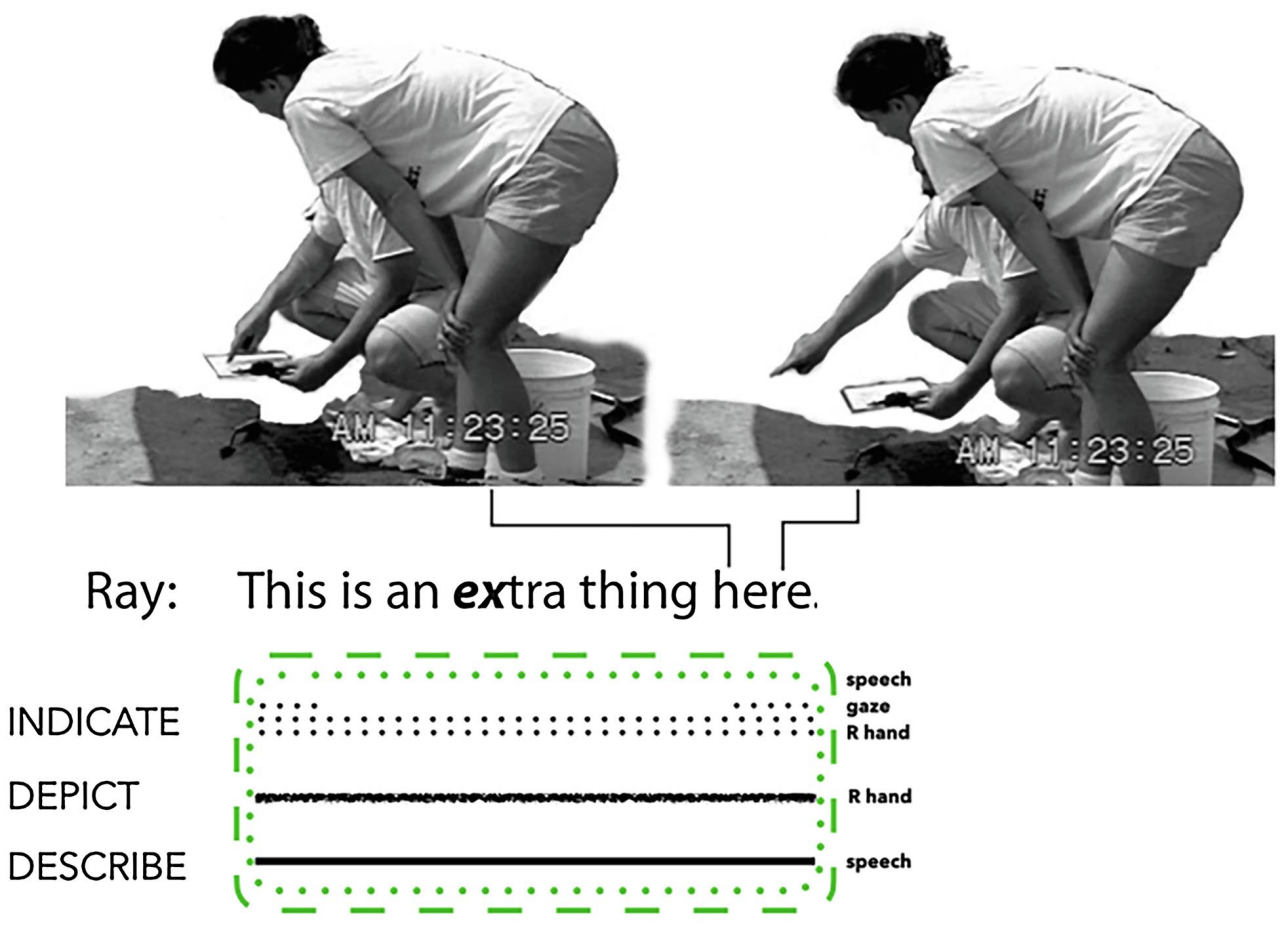
Composite utterance produced by a hearing English speaker (adapted from Goodwin, 2003: 229 and reproduced with permission from Taylor and Francis Group, LLC., a division of Informa plc.).

Goodwin (2003: 14) analyses an interaction during which two hearing archaeologists worked to identify a feature^9^ marked on a map in an area of dirt near them. The speaker says, ‘*This is an extra thing here*’, while simultaneously tracing a little curve on the map with his index finger (see Figure 5). While uttering the final word *here*, the speaker moves his finger to a nearby location on the ground where the feature referred to by *an extra thing* and the tracing movement above the map is visible in the dirt. He then repeats the curved tracing action above this feature within his own line of sight. In this composite utterance, the speaker’s manual tracing action partially depicts the shape of the feature. It also indicates by *directing-to* and *placing-for*: directing others’ attention between the map and the actual feature, and also placing the hand in each location.

This speech and manual action ensemble, produced within the situated participation framework of an archaeological dig, manifest both imagistic and diagrammatic iconicity by depicting (hand), indicating (eye gaze, speech, and hand), and describing (speech). This combination of signalling works to disambiguate the material resemblances of the referents on the map and on the ground. The two one-handed pointing actions used to trace the outline of the feature manifest imagistic iconicity, while the ensemble as a whole exhibits a diagrammatic relation between these two physical map and ground spaces. Indeed, Goodwin analyses this ensemble as an indexical pointing action overlaid on an iconic display. We agree with his conclusion that instead of maintaining a distinction between deictic gestures and iconic gestures, ‘…it seems more fruitful to focus analysis on an *indexical component* or an *iconic component* of a gesture, either or both of which may contribute to the organisation of a particular gesture (Goodwin, 2003: 230, italics in original).

**Figure 6:**
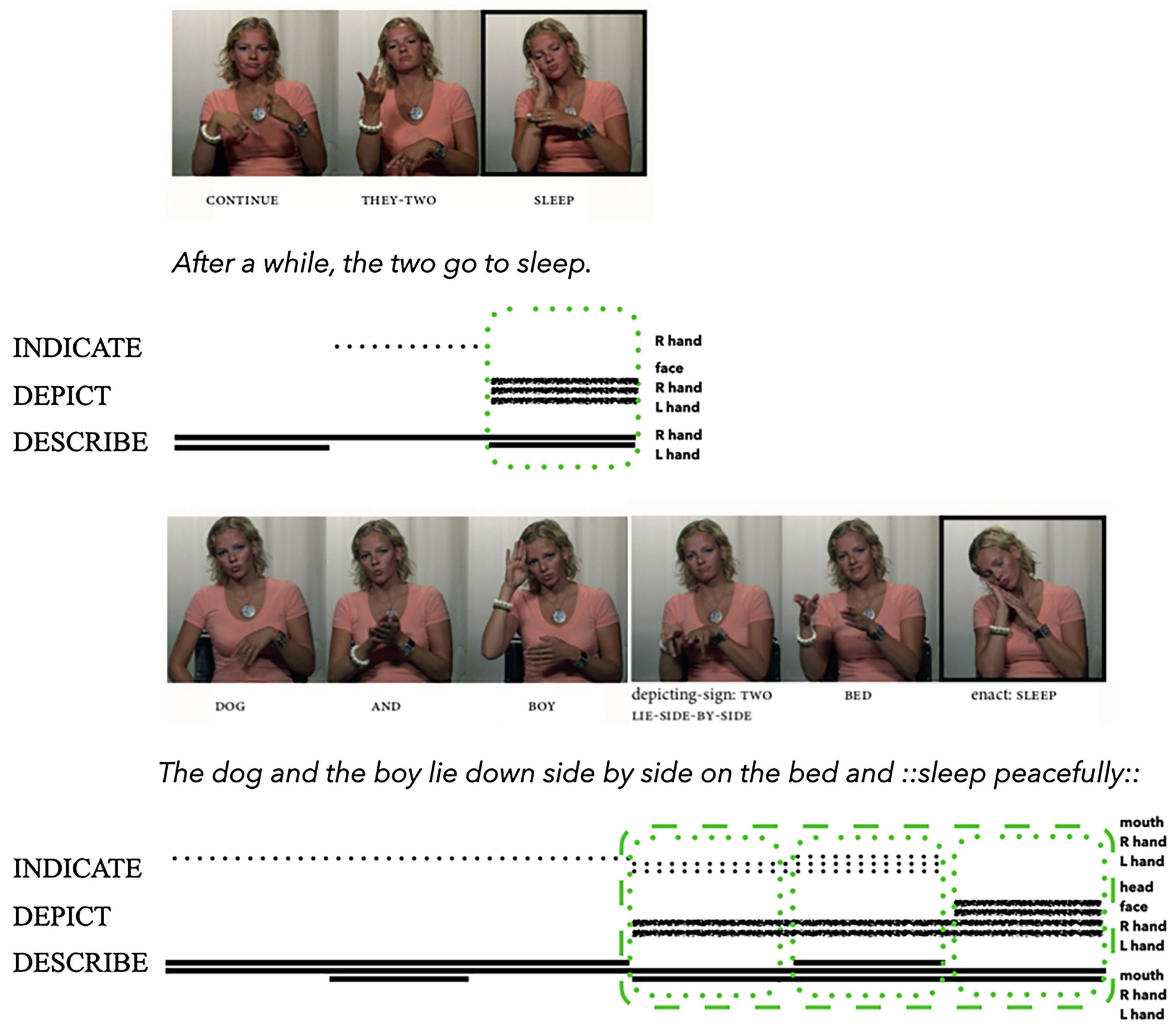
Composite utterance produced by a deaf signer of Norwegian Sign Language (adapted from Ferrara and Halvorsen, 2017: 285 and reproduced with permission from the authors and John Benjamins Publishing Company, Amsterdam/Philadelphia, https://benjamins.com/catalog/gest). This example can be accessed online: Ferrara and Halvorsen, 2021; RPH12_PS_Frosk3.mp4; 00:00:15.39–00:00:20.59.

Signers frequently manipulate the iconic potential of conventionalised manual signs (see Cuxac, 1999; Johnston and Schembri, 1999; Wilcox, 2004; Johnston and Ferrara, 2012; Ferrara and Halvorsen, 2017). While such signs can depict, describe, and/or indicate, the prominence of each signalling method can change (see also Capirci et al., 2022). Ferrara and Halvorsen (2017) analyse two tokens of the sign SLEEP produced by a deaf signer of Norwegian Sign Language across four clause utterances (see Figure 6). Both tokens conventionally symbolise the act of sleeping and therefore describe. Yet as Ferrara and Halvorsen (2017) explain, there are important differences between these two tokens: the signer manipulates the first token to profile a token description and the second token to profile a token depiction. The first token of SLEEP also co-occurs with the mouthing *sove* (*sleep*), which both indexes the spoken Norwegian word and describes this action. This manual sign and mouthed word ensemble result in a ‘double description’ that draws on both Norwegian Sign Language and spoken Norwegian, thus strengthening the descriptive profile of this token. The manual sign also depicts, as the perceptual resemblances between the form (a generalised act of sleeping) and meaning (*sleep*) manifest imagistic iconicity. However, the combined effect is to emphasise the symbolic aspects of this sign and mouthing ensemble: it is an iconic lexical sign instantiating a general type SLEEP, rather than a specific instance of sleeping that is depicted (see also Cuxac and Sallandre, 2007, who refer to this as ‘degenerated iconicity’).

Conversely, the second token SLEEP does not occur with any mouthing. Instead, the signer uses her face and body to emphasise selected visible action qualities of the token instance of sleeping that she wants to depict: the qualities of sleeping deeply and without interruption (see also Balvet and Sallandre, 2014). The second token SLEEP is also framed as a visible re-enactment of an event. While the first token of SLEEP may be analysed as primarily describing the general act of sleeping (describing with two hands and mouth, depicting with hands and face), the second token may be analysed as primarily depicting a specific act of sleeping (depicting with two hands, face, and body, describing with hands). In addition, this second token of SLEEP also occurs as part of a larger multiverb construction (see the sequence of signs: depicting sign:TO-LIE-SIDE-BY-SIDE BED enact:SLEEP in Figure 6). This construction also manifests diagrammatic iconicity, because these forms mirror the sequence of the events in the story, i.e., *the dog and boy lie down side by side on the bed and go to sleep*, and not *the dog and boy go to sleep and lie down side by side on the bed*. By incorporating these details into the analysis, we can better recognise the differences between these iconic forms as they are dynamically instantiated within the interaction.

**Figure 7:**
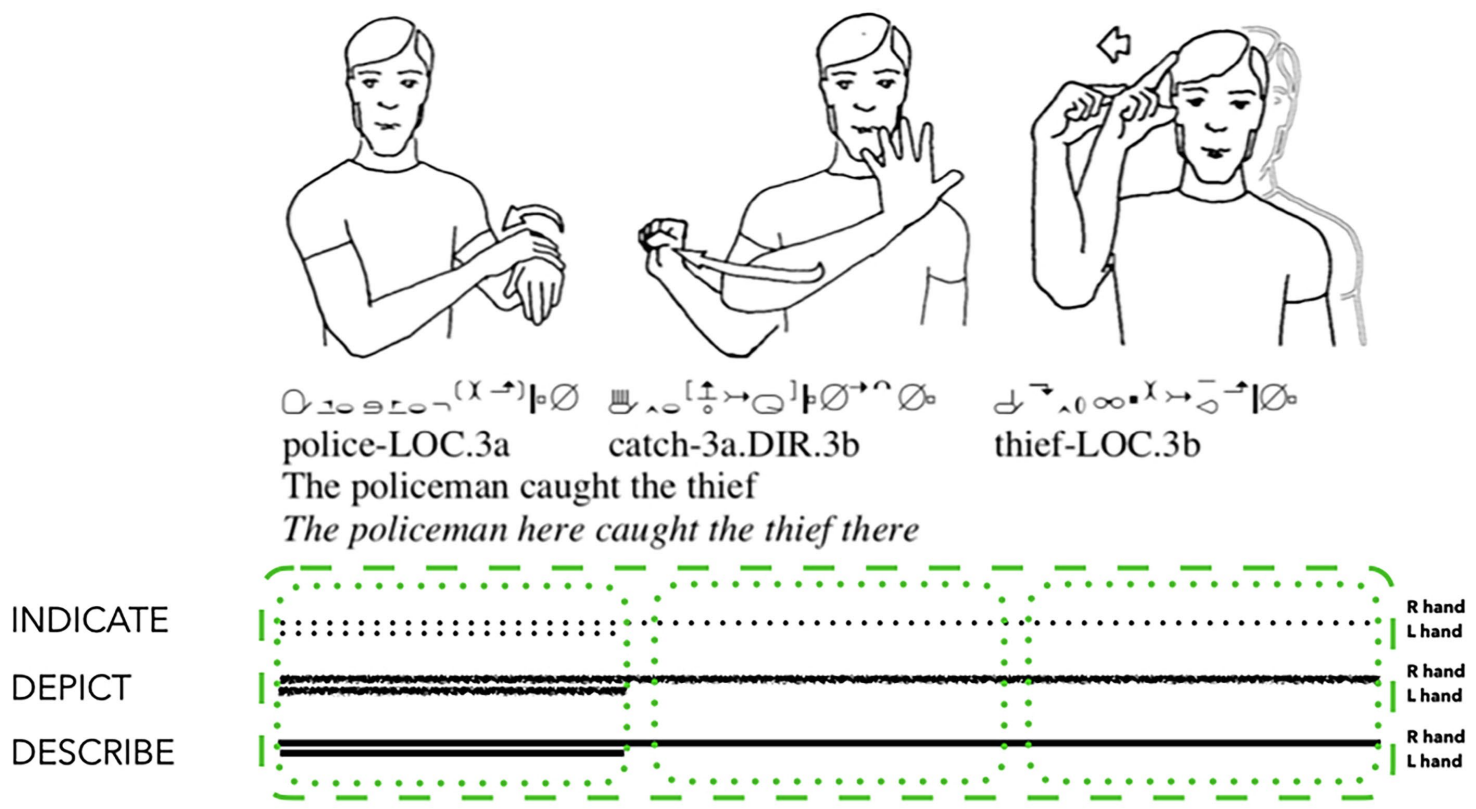
Composite utterance produced by a deaf signer of Auslan (adapted from Johnston, 1996: 72 and reproduced with permission from the author and Taylor and Francis Group, LLC., a division of Informa plc.).

It is also common for signers to manipulate the iconic potential of their immediate spatiotemporal context for syntagmatic reasons (Engberg-Pedersen, 1993; Johnston, 1996; see also De Weerdt, 2020, on the spatiotemporal manifestation of figure-ground relations in FinSL). Johnston (1996) analyses a token of the spatially motivated Auslan utterance POLICE CATCH THIEF (see Figure 7). The three individual sign tokens used in this utterance are all conventionalised Auslan signs, and therefore describe. Each sign also manifests imagistic iconicity signalled through depiction: the sign POLICE resembles the stripes on a policeman’s uniform sleeve and/or the handcuffs used for an arrest; the sign CATCH resembles an act of grabbing a person or object; and the sign THIEF resembles the outline of an imagined thief’s mask. These signs are similar to the first token of SLEEP produced by the Norwegian signer analysed above. Yet there are more schematically iconic aspects of this Auslan utterance in addition to imagistic iconicity. The sequential order and timing of these three signs, along with their meaningful *placing-for* in the signing space and *directing-to* between each signs’ placement, mirror both the spatial and agentive relations between policeman and thief (Johnston, 1996). Thus, these manual signs each manifest imagistic iconicity, primarily through describing, depicting, and indicating, while the utterance as a whole manifests diagrammatic iconicity of location (POLICE on the left, THIEF on the right) and agent-patient relationship (POLICE as agent, THIEF as patient).

**Figure 8:**
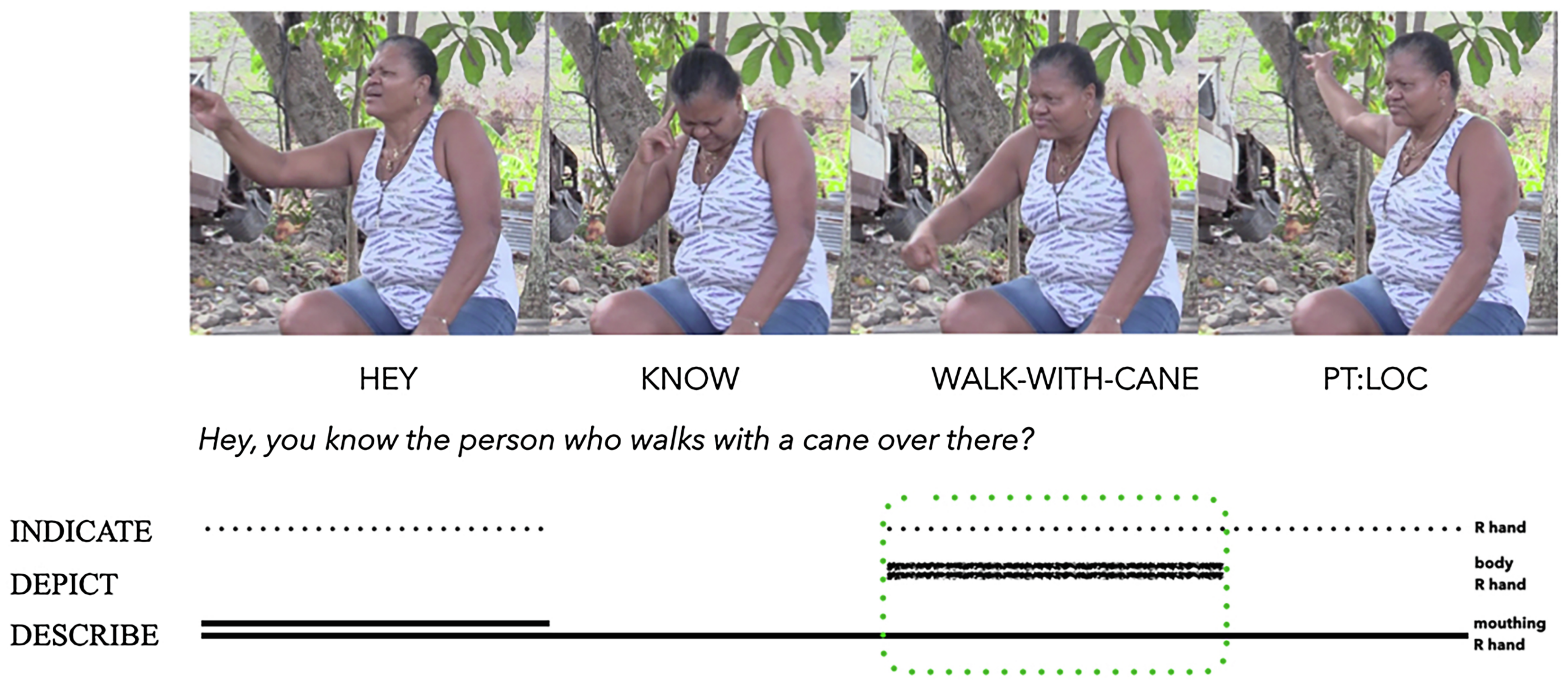
Composite utterance produced by a deaf Providence Island Sign Language (PISL) signer (adapted from Omardeen et al., 2021: 23 and reproduced with permission from the authors and under CC-BY 4.0).

Strategies for depicting in signed interactions may also be used to name referents, in addition to depicting particular qualities of what people, animals, and objects look like or how they move. Omardeen et al. (2021) analyse how deaf signers of Providence Island Sign Language (PISL) use what they term ‘embodied depiction’ for initial person reference. They documented how one PISL signer depicts the specific manner of how another individual walks with a cane, as a way of introducing this non-present person into the discourse (see Figure 8). The signer’s bodily action depicts the visible qualities of the person walking with their cane, while the shape of the signer’s right hand indicates holding the imagined handle of the cane (and hence the cane as an imagined object). As this embodied depiction is conventionally used to refer to a specific individual in the signer’s community, it also describes. In this composite utterance, the signer combines depiction, indication, and description within a manual and bodily action ensemble that manifests imagistic iconicity. The token icon primarily describes a known person into the interaction and discourse context, while also depicting and indicating selected perceptual characteristics of this person. The imagistic iconicity of this ensemble is signalled by depicting (hand and body), indicating (hand) and describing (hand and body) within one composite utterance.

**Figure 9:**
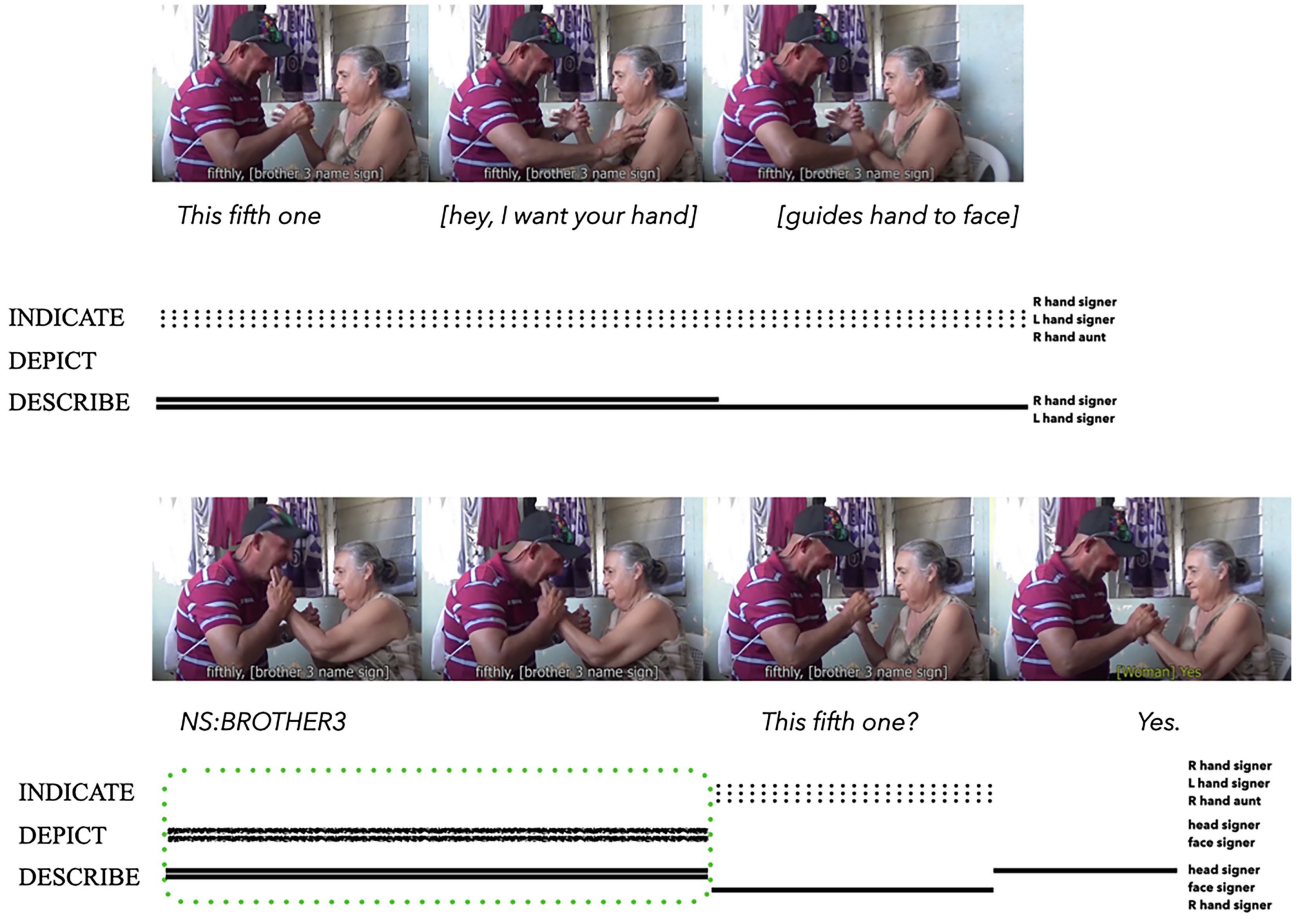
Composite utterance produced by a deafblind BISL signer (adapted from Ali, 2020: 07:18–07:24 and reproduced with permission from the author).

Deafblind signers also make use of iconicity for initial person reference through the tactile co-articulation of bodily actions. Ali (2020) analysed the composite utterances co-articulated by two deafblind signers of Bay Islands Sign Language (see Figure 9). In this instance, the signer on the left is conversing with his aunt on the right. While discussing their family relations, the signer uses his two hands to briefly hold his aunt’s right thumb, thus indexing her fifth and youngest brother. While maintaining this hold, he then guides his aunt’s left hand to his face, so that she can feel him produce the distinctive head nodding movement and facial mannerism that is the conventional name sign of her brother (see the image glossed as NS:BROTHER3 in Figure 9).^10^ In this composite utterance, imagistic iconicity results from depicting (head and face) and describing (head and face) through co-articulation of the tactile name sign ensemble.

**Figure 10:**
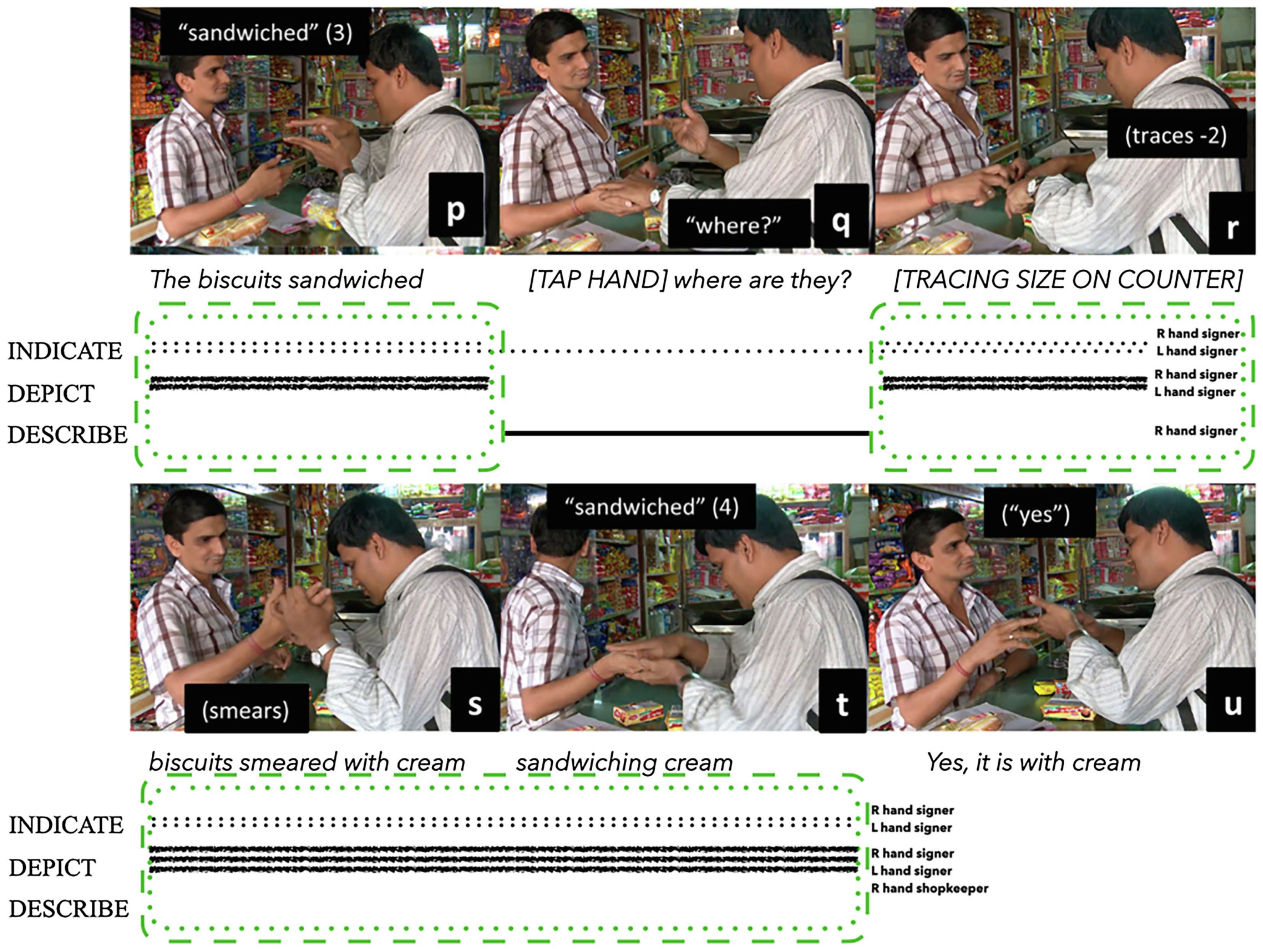
Composite utterance produced by a deafblind Mumbai signer (adapted from Kusters, 2017: 405 and reproduced with permission from the author and Taylor and Francis Group, LLC., a division of Informa plc. The letters **p-u** pertain to the original publication).

Tactile co-articulation of bodily actions is also used between people with sensorial asymmetries, such as deafblind signers and hearing speakers. Kusters (2017) analysed an interaction between a hearing shopkeeper and his customer Pradip, a deafblind man living in Mumbai (see Figure 10). In this example, Pradip is standing in front of a shop counter, behind which the hearing shopkeeper controls what people can see and buy. They have been interacting for some time, as Pradip labours to make himself understood. He wants to buy a specific type of biscuit: cream-filled Marie biscuits. The shopkeeper is closely attuned to Pradip during their interaction, although he sometimes incorrectly guesses or anticipates which type of biscuit Pradip wants. Figure 10 illustrates the moment when the shopkeeper finally understands which biscuits Pradip is asking for. His understanding emerges through three icons that Pradip selectively profiles and co-articulates with the shopkeeper using two of his own hands and the right hand of the shopkeeper: (i) the sandwich shape of the biscuits; (ii) the shape and size of the package the biscuits are sold in; and (iii) the middle of the sandwich biscuits being filled with cream.

Pradip first uses his own two hands to depict the sandwich arrangement of the biscuits he wants, *placing-for* this manual icon where he assumes the shopkeeper can see it (Figure 10p). The shopkeeper turns away but returns with the wrong biscuits (Figure 10q). Pradip then uses his own fingers to trace the shape of the desired package on the surface of the counter, again *placing-for* an outlined depiction of this shape on the counter (Figure 10r). Pradip also gently takes the shopkeepers’ right hand with his own left hand, using his other hand to tactily depict a smearing action on the shopkeepers’ hand, thus beginning the third icon (Figure 10s). Keeping the shopkeeper’s hand held in his own, Pradip then uses his right hand to complete the sandwich depiction, with the shopkeeper’s right hand *placing-for* and co-articulating a depiction of the cream centre of the entire biscuit icon (Figure 10t). Finally, the shopkeeper understands. He turns away and returns with the correct biscuits. He seeks confirmation from Pradip by speaking an utterance combining English and Hindi, and gently pinching Pradip’s left hand (Figure 10u). Pradip can feel the biscuits are the ones he wants and confirms this by nodding his head.

Notably, there is not much describing during this interaction: all propositional information is signalled by combinations of depicting and indicating, especially by *placing-for* on the shop counter. Describing is primarily used for solving problems of understanding during the interaction, as in Pradip’s use of the widely known manual sign WHERE^11^ to request information from a sighted person (Figure 10q), and the shopkeeper’s strategy of gently pinching Pradip’s hands to confirm the biscuits are filled with cream (Figure 10u).^12^ The shopkeeper’s use of describing by speaking English and Hindi to confirm understanding was not heard by Pradip, and therefore not considered integral to Pradip’s interpretation. In these composite utterances, imagistic iconicity is jointly signalled by depicting (Pradip’s two hands and the shopkeeper’s right hand) and indicating (Pradip’s two hands and the shopkeeper’s right hand). Diagrammatic iconicity is also signalled by depicting and indicating (Pradip’s two hands) during the creation of the first icon depicting the sandwich shape of the biscuits, the placement of the second icon on the shop counter, and again during the third icon depicting the cream within the biscuits. Furthermore, the physical presence of the counter heavily influenced the combination of strategies chosen and coordinated by Pradip, as he was observed using different strategies in other interactions that did not involve a shop counter (see Kusters, 2017). This example highlights the importance of sensorial affordances and spatiotemporal contexts for influencing how iconicity manifests during different interactions.

So far we have considered iconic ensembles from a range of co-present interactions. Yet even without moving their hands or body, hearing English speakers make frequent use of iconicity, as evidenced by the prevalence of words such as *sniff, murky,* and *buzzing*, each selectively profiling the different sensorial qualities of various perceptual experiences (Winter et al., 2017). Such words have often been subject to iconicity ratings within decontextualized experimental tasks, with some forms receiving higher ratings than others. For example, Winter et al. (2017) found that speakers of US English rate the words *clank, mushy, whiny, suck*, and *quick* as highly iconic. Forms depicting sound symbolisms (imagistic iconicity) often also depend on systematic arrangements of particular vowels and consonants across many different words in English, e.g., /s/, /z/, and /f/, with specific sounds prompting relational resemblances across networks of words (diagrammatic iconicity). Thus, while these forms are not presented within composite utterances, they may also manifest imagistic and diagrammatic iconicity via depicting (vowels, consonants) and describing (words).

### Comparing Iconicity Across Interactions

We need a way to compare all these different manifestations of iconicity. Recall the two main issues with how iconicity is defined and operationalised in the section ‘Issues With Defining and Operationalising Iconicity’: the prominence of form and the prominence of perceptual resemblances. Our aim here was to mitigate these two issues and encourage more faithful comparisons of iconicity across interactions, modalities, and languages. We did this by asking two questions: (i) how does the interaction signal depicting, indicating, and/or describing? (ii) how does the interaction manifest imagistic, diagrammatic, and/or metaphorical iconicity? By applying a neo-Peircean framework to these interactions, we can interrogate how different types of iconicity were created using different bodily articulators (multimodal) and signalled through different combinations of depicting, indicating, and/or describing (polysemiotic) within single forms and across composite utterances. The issue with the prominence of form is solved by recalibrating analyses of iconicity as signalled polysemiotically within multimodal ensembles. The issue with the prominence of perceptual resemblances (i.e., resemblances of quality) is solved by also considering diagrammatic and metaphorical resemblances (i.e., resemblances of relation and/or association). We can now see how the iconicity identified in these examples all differ in fundamental ways. Crucially, none involve depiction alone. Most rely on more than two articulators, and several manifest one other type of iconicity in addition to imagistic iconicity.

The value of this analysis is evident from just some of the many comparisons that can now be undertaken. Consider, for example, the Siwu speaker and Norwegian Sign Language signer, who both made use of imagistic iconicity in their composite utterances. The Siwu speaker created his multimodal, polysemiotic ‘gunpowder flame’ icon by depicting, indicating (both *placing-for* and *directing-to*) and describing with his two hands and speech. The Norwegian Sign Language signer created her first token of SLEEP by depicting and describing with her two hands and face. She then created her second token of SLEEP by depicting and describing with her hands, head and face, thereby creating an icon that is more closely comparable with manual gunpowder flames depiction than the first token. The second token of SLEEP also manifested diagrammatic iconicity through the sequential multiverb construction depicting the sequence of events as they occurred in the storey. This aspect of the second token further differentiates it from the first token of SLEEP.

Then, there is the hearing archaeologist and the hearing Australian English speaker. Both created speech and manual action ensembles that were analysed as primarily depicting some objects. However, the manual curved tracing action used to depict a feature overlaid on the ground also involved a resemblance of relation between the map and the ground, i.e., diagrammatic iconicity, while the COMPARISON manual action also involved a resemblance of association, i.e., metaphorical iconicity. The manual curved tracing action done by the hearing archaeologist is more comparable to the outline of a packet of biscuits traced by Pradip into the Mumbai shop counter. As a final comparison, consider the token name sign NS:BROTHER3 co-articulated by the Bay Islands Sign Language signers, and the iconic English words mentioned in the final paragraph of the section ‘Analysing Iconicity in Interactions.’ All these tokens depict and describe to create imagistic iconicity, but the Bay Island Sign Language example involves using one signer’s two hands, head and face, and the other signer’s right hand, while the English words in this instance rely on written forms only. In fact, all the examples re-analysed here differ quite substantially from such iconic spoken or written forms used by English speakers. At the very least, tokens that manifest imagistic iconicity through depiction and indication are more comparable with each other than tokens that manifest imagistic iconicity through combinations of depiction, indication and description, although the number of articulators used and the prominence of the different signalling methods is important. As we have demonstrated here, the presence of diagrammatic and/or metaphorical iconicity also needs to be considered.

### Recognising Iconicity as Plurifunctional

The analysis and comparison of how iconicity manifests multimodally and polysemiotically across these interactions prompts a deeper and more pervasive question: why do we do it? Much of the literature has focused on the role of iconicity for human cognition, language development and language evolution, typically by analysing how specific iconic forms are created and used (see the section ‘The Prominence of Form in Analysing Iconicity’). A primary function of iconic ensembles is to show selective qualities of what one means, such as by drawing or performing a picture and/or by creating resemblances of relation and association, rather than describing these qualities through non-resemblances (see Haiman, 1985; Müller, 2014; Clark, 2016). It has been shown that iconicity supports the development of early languaging repertoires and any subsequent language learning (e.g., Imai and Kita, 2014; Perniss and Vigliocco, 2014; Ortega and Morgan, 2015; Ortega, 2017; Nielsen and Dingemanse, 2021). Iconicity helps us figure out what we want to say and how we can say it (McNeill, 1985; Goldin-Meadow, 2003). It enables us to be creative and improvise meaning, and to communicate expressively and efficiently (e.g., Clark, 1996; Kendon, 2004; Fusellier-Souza, 2006; Hodge and Ferrara, 2014; Slonimska et al., 2021). Iconicity is also important for the negotiation and co-regulation of joint actions within social participation frameworks, such as by aligning our manual actions with those of our interactant (Goodwin, 1986; Rasenberg et al., 2020).

Some types of iconicity are fundamental principles explaining language variation and change, while others are merely an effect of how we communicate within specific (socio)linguistic and cultural contexts (see Haspelmath, 2008; Perlman, 2017). For example, imagistic iconicity has been shown to be central to the evolution of displacement in language, supporting the transition of functionally referential signs to conceptually referential signs (Perniss and Vigliocco, 2014). Some communicative strategies that particularly suit the creation of imagistic iconicity may be useful in specific contexts, such as deaf signers’ use of manual depicting actions for talking about referents or processes that do not have a readily available lexical form, or when such a conventionalised form is unknown due to oppressive social and/or educational experiences (Klima and Bellugi, 1979; Fusellier-Souza, 2006; Major et al., 2012; Hodge and Goswell, 2021). Particular types of iconicity can be useful in interactions involving people who have experienced cognitive disruptions such as aphasia (e.g., Neils, 1995; Schveiger, 1995; Wilkinson et al., 2010; Meteyard et al., 2015; Pritchard et al., 2015) and people who are neurodiverse (e.g., Dargue et al., 2021).

These are all valuable lines of investigation, yet there is one more that needs to be considered for the question of ‘why iconicity’: what is the social role of iconicity, and what power does it afford? As with all other aspects of language and communication, it is necessary to consider the broader socio-functional dimensions of iconicity in addition to the semantico-referential ones (see Silverstein, 1976; Halliday and Hasan, 1989; Bernstein, 2003/1971). As Clark observes, ‘How speakers [and signers] make their choices is part of their broader decisions about what they are doing and why’ (1996: 186). Interest in the socially indexical and ‘beyond referential’ aspects of language and communication can be traced back to early scholars concerned with the relationship between people, language and the body politic, or the concept of ‘language as dialogue’ (Vološinov, 1973; Bakhtin, 1981; see Linell, 2009; Spronck, 2019). The basic tenet of dialogism is that all aspects of language are referentially, contextually, and socially grounded (see Gurdin, 1994). This thread was later taken up by others researching the sociology of language use (e.g., Silverstein, 1976; Halliday and Hasan, 1989; Bernstein, 2003/1971). As mentioned in the section ‘Imperatives for Defining and Operationalising Iconicity’, it entails recognising the multilingual and multimodal repertoires that different people and communities develop and draw upon in different contexts and for different (socio)linguistic and cultural reasons (Busch, 2012; Kusters et al., 2017).

In order to consider the social functions of iconicity, we also consider indexicality as a dialectic condition, and not solely a referential strategy. Silverstein (1976) contrasts these two notions of indexicality. He defines referential indexicality as overlapping with referential functions, which were the focus of the analyses presented in the section ‘Analysing Iconicity in Interactions’ (see also Mittelberg, 2008; Kok et al., 2016). He defines non-referential indexicality as signalling elements of the interactional and sociocultural context (i.e., ‘the field, tenor, and mode of discourse’, Halliday and Hasan, 1989). For example, Javanese speakers use deference indexes to stratify interactions between people of high and low social status, and Dyirbal speakers strategically select everyday vs. mother-in-law lexical items to create and maintain sociological distance in relationships (Silverstein, 1976: 32). An example from signed interactions is how experienced Auslan signers might quickly fingerspell full English sentences to other fluent signers in the presence of people who are learning Auslan, both to impart some propositional information about the learner pertinent to their acceptance (or not) within the social context, while excluding comprehension for these learners (see also Tapio, 2014). Such socio-functional aspects of language use can be incorporated into a modality-agnostic, comparative semiotics of iconicity (see also Gurdin, 1994; Radwańska-Williams, 1994).

**Figure 11:**
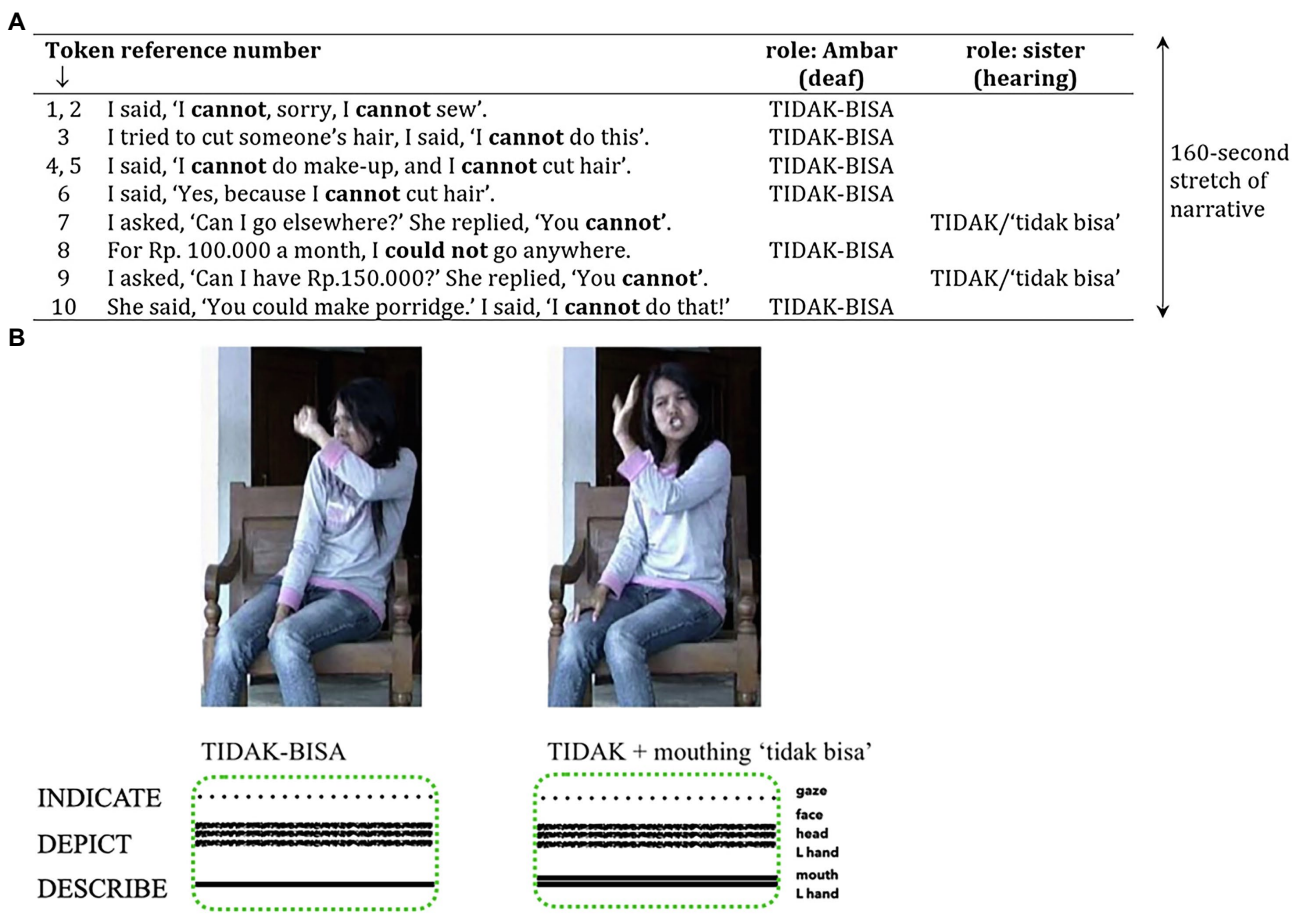
**(A)** English translation of Ambar’s composite utterances (adapted from Palfreyman (2020): 104 and reproduced with permission from the author and John Benjamins Publishing Company, Amsterdam/Philadelphia, https://www.benjamins.com/catalog/aplv). **(B)** Two sign variants for negating the predicate ‘can’ recreated by Ambar (adapted from Palfreyman, 2020: 105 and reproduced with permission from the author and John Benjamins Publishing Company, Amsterdam/Philadelphia, https://www.benjamins.com/catalog/aplv).

Consider the following example from an investigation of the social meanings of variation in BISINDO (Indonesian Sign Language; Palfreyman, 2020; see Figure 11). Here, a young deaf signer Ambar is talking to a deaf friend about her experiences of trying different professions before finding a suitable job. Figure 11A provides an English translation of how Ambar recreated an earlier conversation between herself and her elder hearing sister, using visible bodily enactment (i.e., ‘personal transfer’, ‘constructed action’, ‘reported speech’) to depict these earlier utterances. Each utterance involved one of two different variants for negating the predicate ‘*can*’. Ambar uses the variant TIDAK-BISA for her own utterances, and another variant TIDAK + mouthing ‘*tidak bisa*’ for those of her hearing elder sister (see Figure 11B). The two variants impart different social meanings: TIDAK-BISA is a suppletive manual sign that is commonly used by younger deaf signers from the Solo (Central Java) region, whereas the variant TIDAK + mouthing ‘*tidak bisa*’ has its origins in the manual gestures and Indonesian mouthings used by hearing non-signing speakers. There is an implicit BISINDO ideology that ‘the suppletive variant is more “deaf” than the mouthed predicate construction, which is more “hearing” because of its gestural associations’ (Palfreyman, 2020: 15).

In these composite utterances, Ambar combines depicting, indicating and describing within manual and bodily action ensembles to enact the utterances (and negation variants) previously used by herself and her sister. Altogether, the TIDAK-BISA ensemble manifests imagistic iconicity by depicting (face, head, and hand), indicating (gaze) and describing (hand), while the TIDAK + mouthing ‘*tidak bisa*’ ensemble manifests imagistic iconicity by depicting (face, head, and hand), indicating (gaze) and describing (hand and mouthing). Ambar therefore uses these iconic bodily enactments to reference who is saying what to whom (Ambar; her elder sister), while simultaneously indexing the different social roles of each person, and local ideologies regarding their chosen communication practices (a young deaf local signer; an older, hearing sibling who does not know BISINDO). Thus, Ambar also communicates her epistemic evaluation of the previous conversation between herself and her sister, whereby she selectively imbues her personal values into the depiction to take a stance about it (see Niemelä, 2010). In terms of the participation framework in which these composite utterances unfold, these enactments aid Ambar to ‘other’ her sister as a hearing outsider within deaf social contexts. Thus, the imagistic reconstruction of a prior conversation indexes both the referential aspects of the interaction and the social dynamics of the people involved, through the lens of one of those people. This aspect of iconicity is vital in every sense of the word, but is often masked in experimental investigations.

There are many socio-functional dimensions of iconicity present in the examples analysed above. Consider the POLICE CATCH THIEF example in Figure 7. Johnston (1996) actually provided eight variations of this propositional utterance, all of which vary in the order of signs and/or meaningful use of space. The example chosen for our purposes here most closely reflects the choices made by experienced and highly respected Auslan signers who are proficient in making meaningful use of space. Thus, this particular construction also indexes specific Auslan socialities: people who have signed since birth or early childhood, or who have otherwise experienced maximal opportunities to sign this way (see Hodge and Goswell, 2021). Then, there is the Alice Springs air writing example in Figure 3, which indexes the development of English literacy practices used by young and older people in Ngaanyatjarra communities (Ellis et al., 2019: 105). The embodied depictions used by the Providence Island signer (Figure 8) and the deafblind Bay Islands signers (Figure 9) index specific sociocultural norms regarding how people are physically perceived and known, and how they are identified and named.

Finally, there are the iconic ensembles co-articulated by Pradip and the Mumbai shopkeeper in Figure 10. These icons are more restricted in terms of non-referential indexicality, since so much effort is invested in establishing referential common ground, but look closely and it is there: in Pradip’s expert labouring and strategic use of iconicity in building mutual understanding, to achieve self-determination and personal agency by connecting directly with someone who has a vastly different sensory embodiment, rather than indirectly through a ‘helper’ who can rely heavily on describing, such as a signed language interpreter (see also Clark, 2021; Moriarty and Kusters, 2021; Green, 2022). Herein lies the social role of iconicity, and the power it affords: we use iconicity to index our relationships, our experiences, and our socialities. We use it to live our lives. It is therefore just as important to consider the socio-functional aspects of iconicity as the semantico-referential aspects, since much depends on the people interacting and the resources available within specific social and spatiotemporal contexts (see also Sicoli, 2010).^13^

This has implications for how we can expand discussions of iconicity across the language and communication sciences. For example, researchers have highlighted the important role of depicting for efficient referential communication between signers who share a signed language (e.g., Slonimska et al., 2021). Yet when we consider how iconicity manifests between people with sensorial asymmetries such as Pradip and the shopkeeper, it becomes apparent how much effort and labour is often involved in signalling through depicting. This highlights a moral aspect to using iconicity: it can also reflect people’s willingness to both understand and make oneself understood, especially during interactions when people must rely on ‘far leaner linguistic resources than users of conventional languages’ (Green, 2022: 22; see also Goodwin, 1995; Moriarty and Kusters, 2021). Thus, the socio-functional role of iconicity may also change according to the people interacting and the sociocultural context. Table 1 summarises some multimodal, polysemiotic, and plurifunctional dimensions of the examples analysed here.

**Table 1:** Iconicity as multimodal, polysemiotic, and plurifunctional (number of articulators in parentheses and note this summary is not necessarily exhaustive).

	Multimodal	Polysemiotic	Plurifunctional	Iconicity
Hissing, buzzing (English)	Speech (1)	DD	Referential	Imagistic
Tree (ASL)	Hands (2)	DD	Referential	Imagistic
To sleep (Norwegian SL)	Hands, face, and mouthing (4)	DD	Referential	Imagistic
Younger brother (tactile BISL)	Head, face (2)	DD	Referential, social	Imagistic
Sleeping deeply (Norwegian SL)	Hands, face, head (4)	DD	Referential, social	Imagistic, diagrammatic
Biscuit sandwich (tactile signs)	Hands (2)	DI	Referential, social	Imagistic, diagrammatic
Biscuit package (tactile signs, counter)	Hands (2), object (1)	DI	Referential, social	Imagistic, diagrammatic
Biscuit contents (tactile signs)	Hands, other hand (3)	DI	Referential, social	Imagistic, diagrammatic
Alice Springs (Ngaanyatjarra, English)	Hand, speech (2)	DID	Referential, social	Imagistic
Person with cane (PISL)	Hand, body (2)	DID	Referential, social	Imagistic
Catching thief (Auslan)	Hands (2)	DID	Referential, social	Imagistic, diagrammatic
An extra thing (English)	Gaze, hand, and speech (3)	DID	Referential, social	Imagistic, diagrammatic
Gunpowder flames (Siwu)	Hands, speech (3)	DID	Referential, social	Imagistic
Working it out (English)	Gaze, hands, and speech (4)	DID	Referential, social	Imagistic, diagrammatic, metaphorical
TIDAK-BISA (BISINDO)	face, gaze, hand, head (4)	DID	Referential, social	Imagistic
TIDAK + tidak bisa (Indonesian)	Face, gaze, hand, head, and mouthing (5)	DID	Referential, social	Imagistic

## Discussion

In this paper, we reconceptualised and operationalised iconicity as multimodal, polysemiotic, and plurifunctional. We end by discussing some implications for the language and communication sciences, and explain how this approach guides us towards a theory of biosemiotics. It is first necessary to assess if this framework is useful and effective. Using Occam’s Razor, we determined six criteria against which the framework can be assessed in terms of its explanatory power (see Hossenfelder, 2020): (1) The framework must be able to account for the full range of iconicity observed across human interactions, not just hearing, able-bodied interactions; (2) It must align with known principles explaining language and communication more generally, or at least not contradict them; (3) It must enable continuity across different time frames, e.g., enchrony, synchrony, and diachrony; (4) It must be operationalisable using transdisciplinary methods, e.g., available for experimental methods, corpus annotation, language assessment, pedagogy; (5) It must enable continuity and comparability with nonhuman communication, and compatibility with other life sciences; and (6) It must make us rethink existing paradigms and consider new ones.

So how does our proposal hold up to this assessment? The analyses presented in the section ‘Analysing Iconicity in Interactions’ demonstrates the framework outlined here does effectively facilitate the modality-agnostic analysis and comparison of iconicity within and across a range of human interactions (1, 3). It does this without marginalising or pathologising anyone, and includes consideration of both semantico-referential and socio-functional aspects of communication (1, 2). The theoretical foundations were established by considering what is known about complex ontogenies of semiosis, language, and communication, as well as broader principles influencing and explaining language variation and change (2, 3). The framework offers tools for quantitative analysis, such as diagnostics for identifying how people depict, indicate, and/or describe; identifying how imagistic, diagrammatic, and/or metaphoric iconicity is manifested; and coding methods that are transferable into machine-readable annotation systems. For example, we have used dotted, dashed, and sharp lines or boxes here for ease of illustration, but this coding schema could easily be operationalised as tiers within time-aligned video annotation software such as ELAN. It also offers tools for qualitative analysis, such as consideration of the sociocultural aspects of specific interactions and how these might influence people’s choices for manifesting iconicity, including and beyond any immediate need to establish referential common ground. The framework can therefore be operationalised by researchers using a range of methods (1, 4). But what about continuity with nonhuman communication? The need to strive for a science that unifies the destructive schisms between humans and nature is important to us (5).

It was polymath Thomas Sebeok (1920–2001) who suggested that ‘life and semiosis are coextensive’, a concept he developed by looking for evidence of semiosis across the life sciences, especially across the animal world (see Barbieri, 2009, for an overview). His insights played a large part in the unification of semiotics and biology—*biosemiotics*—the main purpose of which is to show that ‘signs and meaning exist between all living systems’ and that ‘semiosis is a fundamental component of life’ (Barbieri, 2009: 222; see also Deacon, 1997; Favareau, 2015). There is not enough space here to do justice to such a broad and relatively new field, suffice to say that we can draw on Sebeok’s approach by asking not what makes iconicity different from nonhuman communication (or the traditional preoccupation with what makes *arbitrary symbols* different). Rather, we ask what makes it the same (see also Perlman, 2017).

The question of whether or not gorillas, for example, use iconicity is a matter of great debate (see Perlman et al., 2014). Perlman and Gibbs (2013) describe the ‘iconic gestures’ used by Koko, a human-fostered gorilla, with the aim of determining if her token gestures suggest a sensori-motor imagery similar to humans. Five tokens of iconic gestures identified within a corpus of video-recorded interactions between Koko and her two main human caregivers were analysed. All involved re-enactments of embodied actions (‘pantomimes’) that Koko wanted her caregivers to perform, such using a set of keys to act out unlocking a door (to request an outside walk), or acting out wiping a pair of sunglasses with an imaginary tissue (to request a Kleenex). Perlman and Gibbs (2013) argue these actions were clearly produced for communicative purposes, as they were different in force and effect to how Koko would produce them for instrumental purposes. For example, a back scratch gesture done with instrumental force (to scratch an itch) appeared different to a back scratch done with communicative intent (to request a caregiver scratch a different place on her back). Several actions were also novel or obviously tailored for the specific context. Similar actions have also been observed during interactions between free-ranging chimpanzees (Pika and Mitani, 2006). Perlman and Gibbs (2013) suggest these actions constitute iconic gestures, and we agree with them: with the additional suggestion that Koko’s embodied actions could be interpreted as different combinations of depicting, indicating, and describing developed throughout her lifelong experiences of interacting with her human caregivers.

Indeed, such a use of this framework may not be restricted to analyses of iconicity; it could also extend to nonhuman referential indexicality. For example, Vail et al. (2013) describe the ‘referential gestures’ used by some coral reef fish (groupers and trout) to ‘indicate’ the presence and location of hidden prey to cooperative hunting partners such as giant moray eels and Napoleon wrasses. Groupers were observed to use two different signals to initiate and coordinate collaborative hunts with moray eels: (i) a high frequency and horizontal body shimmy that is performed in front of a sheltering moray, which results in the moray accompanying the grouper on a collaborative food hunt; (ii) a vertical, headstand orientation produced with headshakes that have pauses between them, placed over a narrow crevice in which escaped prey fish are hiding, which sometimes resulted in the slender moray eel darting into the crevice to hunt the prey, a possibility not available to groupers since they are too large. The authors suggest these signalling actions share the hallmarks of intentionality, and we agree with them: with the additional suggestion that the indicating signals used by these fish could be interpreted as involving both *directing-to* and *placing-for*.

In other words, it is not a huge stretch to consider that Koko’s use of keys to poke at the lock in the door, or use of her fingers to demonstrably scratch her back, might be interpreted as an ‘icon’ by another human or gorilla, or that the placement of a grouper over a narrow crevice in the context of a collaborative hunt might be interpreted as an ‘index’ by a moray eel. However, it is obviously a problem if we attribute definitive human interpretations to the possibilities experienced by gorillas, fish and eels within their own umwelts. The main point we want to make here is that the communicative behaviours observed within these cross-species interactions are contiguous with human pathways for signalling through indicating, depicting, and/or describing (5).

Finally, the framework proposed here does make us rethink existing paradigms, simply by the questions it asks us to answer: (i) how do we combine depicting, indicating and/or describing within an interaction? (ii) how does the interaction manifest imagistic, diagrammatic and/or metaphorical iconicity? To interrogate these questions, it is necessary to initially focus on interactions (not individuals) and situated contexts (not languages; see also Kusters et al., 2017). Then, there is the process of analysing, annotating, and comparing iconicity within and across interactions (see the sections ‘Analysing Iconicity in Interactions’ and ‘Comparing Iconicity Across Interactions’). After observing how often speakers make use of improvised bodily actions that are tightly coordinated with conventionalised speech or how often signed depictions also describe, does it still make sense to operationalise binaries such as ‘signers vs. speakers’, ‘words vs. signs’, ‘spoken languages vs. signed languages’, ‘verbal modality vs. gestural modality’, or even ‘convention vs. improvisation’ in experimental methods or language theory? Does it still make sense to credit the ease and efficiency of ‘drawing a picture’ as the main motivation for manifesting iconicity, or can we now consider there may be other, more subterranean forces related to human sociality? As we move further along the path of comparative semiotics, it may be useful to question whether these paradigms continue to serve our understanding in a progressive way. Perhaps some are better characterised as intellectual conveniences (and historically, political necessities) that we can gradually do without. For this reason, we add an overarching coda to the method outlined in the section ‘Recognising Iconicity as Multimodal and Polysemiotic’: (iii) why are the people in the interaction communicating like this? We may not always discover the answer, but we should certainly ask the question.

There are two broader implications for the language and communication sciences. Firstly, iconicity is more complicated than how it is often conceptualised and operationalised in the literature. This complexity needs to be recognised and accounted for within empirical methods and the interpretation of findings relating to iconicity in language and communication. For example, it is not sufficient to propose that one is ‘investigating iconicity’—we need to be specific about what kinds and how it manifests. Secondly, particular thought needs to be given to how the indicating and describing signals of an iconic ensemble may affect the interpretation of results and findings from experimental and other studies. Finally, and perhaps most importantly, deeper consideration of the social functions of iconicity may offer richer or even better explanations for why we do it. This may lead to the reanalysis of some prior claims, while others may be better supported, but at least we will be able to address some of the biases described earlier and compare like with like.

## Conclusion

In this paper, we argued that iconicity is multimodal, polysemiotic, and plurifunctional. By applying the theory of language use outlined by Clark (1996) to a range of different interactions, and also considering the notion of non-referential indexicality proposed by Silverstein (1976), we illustrated the multidimensionality of iconicity as emerging through the creation of different types of icons, all of which are minimally signalled by depicting, but usually also with indicating and/or describing, and usually with more than one bodily articulator. Analyses from a range of co-present interactions highlight how iconicity often emerges across larger ensembles of joint multimodal actions, in addition to smaller units such as words and signs, all of which can range from concrete to more schematic. These analyses also highlight how imagistic, diagrammatic and/or metaphorical iconicity may manifest within these ensembles. This framework facilitates a more accurate analysis and comparison of iconicity across interactions, modes of communication, and languages. It also facilitates consideration of the question of why we do it, from referential functions through to social functions. By reconceptualising and operationalising iconicity in this way, we can do justice to human social complexity in our efforts to understand how languaging works and why it differs, while advancing possibilities for a modality-agnostic comparative semiotics that is not limited to our human domains.

## Data Availability Statement

The original contributions presented in the study are included in the article. Further inquiries can be directed to the corresponding author.

## Ethics Statement

Written informed consent was obtained from the individual(s) for the publication of any potentially identifiable images or data included in this article.

## Author Contributions

GH and LF conceptualised the study, undertook the literature review, analysed the examples, and detailed the theoretical argumentation. GH wrote 60% of the manuscript. LF wrote 40%. All authors contributed to the article and approved the submitted version.

## Funding

GH acknowledges support from the United Kingdom Arts and Humanities Research Council (AH/N00924X/1) and the Economic and Social Research Council (ES/R011869/1). LF acknowledges support from the Norwegian Research Council (Project no. 287067).

## Conflict of Interest

The authors declare that the research was conducted in the absence of any commercial or financial relationships that could be construed as a potential conflict of interest.

## Publisher’s Note

All claims expressed in this article are solely those of the authors and do not necessarily represent those of their affiliated organizations, or those of the publisher, the editors and the reviewers. Any product that may be evaluated in this article, or claim that may be made by its manufacturer, is not guaranteed or endorsed by the publisher.

## Footnotes

^1^ https://bslsignbank.ucl.ac.uk/dictionary/words/you-2.html

^2^ https://aslsignbank.haskins.yale.edu/dictionary/gloss/342.html

^3^ https://aslsignbank.haskins.yale.edu/dictionary/gloss/812.html

^4^ https://aslsignbank.haskins.yale.edu/dictionary/gloss/1148.html

^5^ https://aslsignbank.haskins.yale.edu/dictionary/gloss/1237.html

^6^ https://auslan.org.au/dictionary/words/give-1.html

^7^Note that others use the term *polysemiosis* to refer to combinations of different articulations or forms of communication, such as how pantomime might involve combinations of bodily gestures, vocalisations and facial expressions (e.g., Zlatev et al., 2020). This is fundamentally different to the definition of *polysemiosis* used here. Indeed, Zlatev and colleagues’ definition is perhaps closer to our use of the term *multimodal*. Our aim here is to address issues with the prominence of form in studies of iconicity (see the section The Prominence of Form in Analysing Iconicity) and to encourage identification of similarities across human and nonhuman communication, not just differences (see the section Discussion).

^8^It would be somewhat misleading to suggest that each mode of signalling is either ‘present’ or ‘absent’, because in Peircean terms, all three modes are always present in more or less degrees. In this sense, each instantiated token is also an icon of any previous instantiations. For example, a token finger-pointing action that indexes a location is also an icon of the type ‘indexical actions pointing to a location’. We do not address this level of analysis in the current paper, but it is something to keep in mind.

^9^An indication of some non-portable human activity, such as a black stain indicating the cinders of a hearth.

^10^The nephew earlier produced four name signs for the other siblings on his aunt’s face, as each name sign involves some form of external tactile touch and movement, such as tapping or gently pinching (see Ali, 2020). However, the younger brother’s name sign involves movements that can only be done by the person who is signing, since it is not socially acceptable to forcibly move or contort a co-articulator’s head or face in the way required (at least not in the way it is possible to do with arms and fingers). Thus, this rich example also highlights the intersubjective norms for co-articulated communication regarding whose body is recruited for what actions at any given moment (see Edwards, 2015; Willoughby et al., 2020; Clark, 2021). This point is relevant to understanding the different affordances that influence people’s use of iconicity in different contexts.

^11^This sign is not exclusively a deaf sign, it is widely known and used by hearing people in Mumbai.

^12^Indeed, the interactional strategies of turn-taking and repairs choreographed by Pradip, and which scaffolded the co-articulation of the three different biscuit icons, are more ‘conventional’ than the content of what was said, in the sense that Pradip initiates these repairs turn-by-turn and the shopkeeper responds to them by (mis)understanding (see the section ‘Signalling by Describing’).

^13^Sicoli (2014) later observed that Peirce’s most well-known trichotomy of *symbols, indices, icons* may not be the most appropriate tool for analysing iconicity, and that his trichotomy of *rheme, dicent, argument* better highlights the ‘joint activity’ of languaging in terms of its performativity, recipient design, and interpretability. We agree this is a solid proposal for future consideration, and hope that eventually these frameworks may be united.
